# A Multiplex Genome Editing Method for *Escherichia coli* Based on CRISPR-Cas12a

**DOI:** 10.3389/fmicb.2018.02307

**Published:** 2018-10-09

**Authors:** Xiang Ao, Yi Yao, Tian Li, Ting-Ting Yang, Xu Dong, Ze-Tong Zheng, Guo-Qiang Chen, Qiong Wu, Yingying Guo

**Affiliations:** ^1^MOE Key Laboratory of Bioinformatics, Center for Synthetic and Systems Biology, Tsinghua University, Beijing, China; ^2^Tsinghua-Peking Center for Life Sciences, Tsinghua University, Beijing, China; ^3^School of Life Sciences, Tsinghua University, Beijing, China; ^4^Center for Synthetic and Systems Biology, Tsinghua University, Beijing, China; ^5^China National Center for Biotechnology Development, Beijing, China; ^6^State Key Laboratory of Environmental Chemistry and Ecotoxicology, Research Center for Eco-Environmental Sciences, Chinese Academy of Sciences, Beijing, China

**Keywords:** CRISPR-Cas12a, synthetic biology, multiplex genome editing, *E. coli*, *Halomonas*

## Abstract

Various methods for editing specific sites in the *Escherichia coli* chromosome are available, and gene-size (∼1 kb) integration into a single site or to introduce deletions, short insertions or point mutations into multiple sites can be conducted in a short period of time. However, a method for rapidly integrating multiple gene-size sequences into different sites has not been developed yet. Here, we describe a method and plasmid system that makes it possible to simultaneously insert genes into multiple specific loci of the *E. coli* genome without the need for chromosomal markers. The method uses a CRISPR-Cas12a system to eliminate unmodified cells by double-stranded DNA cleavage in conjunction with the phage-derived λ-Red recombinases to facilitate recombination between the chromosome and the donor DNA. We achieved the insertion of up to 3 heterologous genes in one round of recombination and selection. To demonstrate the practical application of this gene-insertion method, we constructed a recombinant *E. coli* producing an industrially useful chemical, 5-aminolevulinic acid (ALA), with high-yield. Moreover, a similar two-plasmid system was built to edit the genome of the extremophile *Halomonas bluephagenesis*.

## Introduction

Efficient methods for the introduction of heterologous genes into microbial hosts are indispensable for metabolic engineering and industrial strain construction, and a number of techniques have been developed to provide easy ways to introduce gene insertions or deletions into the genome of *Escherichia coli*. Prominent examples include group II intron retro-homing (Karberg et al., 2001; Enyeart et al., 2013) and recombination-mediated genetic engineering (recombineering) (Datsenko and Wanner, 2000; Heermann et al., 2008; Sharan et al., 2009). Recombineering, in particular, is commonly used for precise editing of the *E. coli* genome. With the assistance of phage-derived recombinases (λ-Red and RecET), efficient DNA integration can be accomplished through recombination between donor DNA and the chromosome at a specific, pre-defined site. However, this process requires the presence of a selectable marker to counter-select the wild-type strain (Yu et al., 2008; Yang et al., 2014), and therefore also necessitates a further step to remove the marker, leaving behind a scar site in some cases (Sukhija et al., 2012; Esvelt and Wang, 2013). As a consequence, for multiplex genome engineering, these methods are time-consuming. Multiplex automated genome engineering (MAGE) (Wang et al., 2009) and co-selection MAGE were developed to perform genomic manipulation through point-mutations or (and) short insertions, but both methods are not suitable for performing gene-size (about 1 kb) insertions.

Recently, the clustered regularly interspaced short palindromic repeats (CRISPR)/CRISPR-associated protein (Cas) system (Mojica et al., 2005; Jiang et al., 2013) has been coupled with the λ-Red system to accomplish efficient editing of the *E.* *coli* genome (Jiang et al., 2015; Li et al., 2015; Pyne et al., 2015; Reisch and Prather, 2015; Zhao et al., 2016; Chung et al., 2017; Zhang et al., 2017). In such methods, double stranded DNA cleavage by the CRISPR-Cas system is used to counter-select against wild-type cells (Chayot et al., 2010). The CRISPR-based selection strategy therefore enables rapid and scarless genomic editing. However, even though some groups achieved simultaneous modifications of up to three genes (Jiang et al., 2015; Li et al., 2015), gene insertions at multiple loci were not performed. In a different approach, Bassalo et al. (2016) developed a strategy to integrate large metabolic pathways into the *E. coli* genome at a single locus. However, when the integrated pathway was further edited *in vivo*, deletions across the targeted site were observed frequently, suggesting recombination between repetitive elements (e.g., promoters and terminators). Therefore, a promising solution, for further manipulation of the genome, is to divide the metabolic pathway into several parts and insert these components into different sites.

Lately, a novel type V-A CRISPR-Cas system - CRISPR-Cas12a (CRISPR-Cpf1) (Zetsche et al., 2015), was described. Distinct from CRISPR-associated protein 9 (Cas9), Cas12a is a single RNA-guided endonuclease, which utilizes a different protospacer-adjacent motif (PAM) and leaves sticky ends after DNA cleavage (Zetsche et al., 2015). Compared with the commonly used *Streptococcus pyogenes* Cas9 (SpCas9), the *Francisella novicida* Cas12a (FnCas12a) harnessed in this research has a smaller size, follows the guidance of a dual CRISPR RNA (crRNA), and utilizes a T-rich PAM (Zetsche et al., 2015). The smaller size of Cas12a decreases the metabolic burden imposed on the host cells, and makes it easier for researchers to handle the corresponding material (e.g., in plasmid construction, electroporation, etc.). The CRISPR-Cas12a system has been adopted for genome editing in several bacterial species, including *Corynebacterium glutamicum* (Yu et al., 2017), *E*. *coli, Yersinia pestis*, and *Mycobacterium smegmatis* (Yan et al., 2017). Although genomic manipulation at a single site was achieved in *E. coli*, an efficient method that can be used to simultaneously perform multiplex gene insertions is needed to achieve time and cost savings.

Here, we describe a rapid and efficient method to edit the *E. coli* chromosome at multiple sites simultaneously and a recombinant *E. coli* integrated with three heterologous genes was obtained within 8 days. By simultaneously integrating the *T7 RNA polymerase* gene and the T7 promoter-driven ALA synthase gene into two separate loci, this system was employed to construct a strain for the efficient production of an industrially useful chemical – ALA (Liu et al., 2014). In addition, the modification of the atypical extremophilic host *Halomonas* using CRISPR-Cas9 (Qin et al., 2018) demonstrates the power of gene editing in different bacterial species. To test the potential of this method in editing other types of bacterial genomes, a similar two-plasmid system based on CRISPR-Cas12a was built to edit the genome of the extremophile *H. bluephagenesis*.

## Results

### Construction of the Two-Plasmid System

**FIGURE 1:**
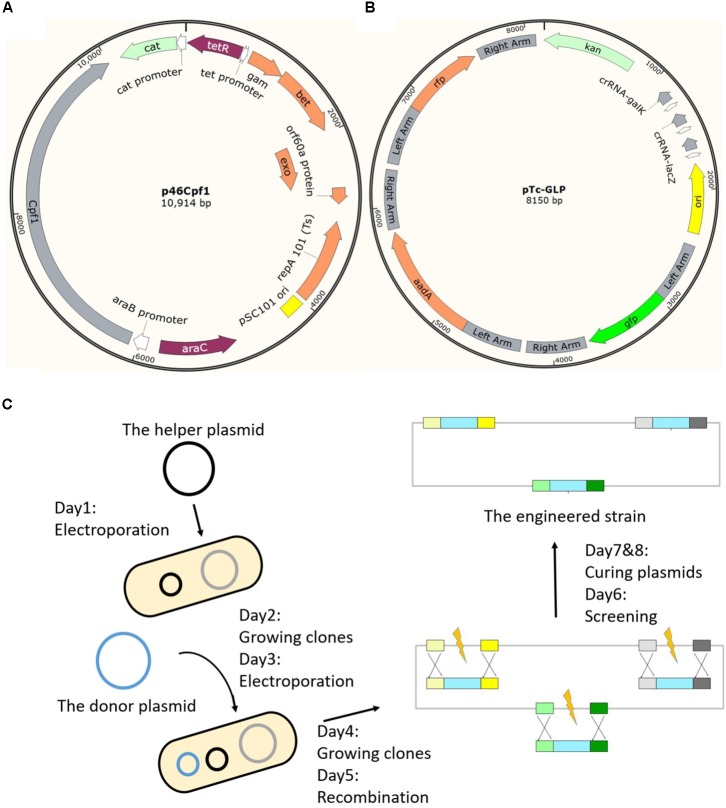
Schematic maps of the developed method. **(A)** A schematic of the helper plasmid p46Cpf1, in which λ Red recombinases (Gam, Bet, and Exo) are expressed under the control of P_tet_, and Cas12a is expressed under the control of P_araB_. **(B)** A schematic of the donor plasmid pTc-GLP, which encodes crRNAs constitutively and provides the templates for recombination at three loci (*galK, lacZ*, and *pyrF*). **(C)** General outline of the multiplex gene insertion method. It takes 8 days to construct a modified plasmid-free strain.

The genome editing method uses a CRISPR-Cas12a system, including Cas12a and corresponding crRNA(s), to eliminate unmodified wild-type cells by double-stranded DNA cleavage, in conjunction with the phage-derived λ-Red recombinases to facilitate recombination between the chromosome and the donor DNA. With CRISPR-Cas12a-mediated restriction to eliminate unmodified cells, homologous sequences in the donor plasmid as templates, and λ-Red to accelerate recombination, we assumed to achieve genomic modifications when all conditions were met. It is worth mentioning that, differing from linear templates (PCR products or DNA oligos), circular templates (donor DNAs in high copy-number plasmids with the pUC origin) were adopted in this study to increase their concentration. We believed that the increase in template concentration should make multiplex engineering possible. The two-plasmid system was composed of a helper plasmid and a donor plasmid (**Table 1**). The helper plasmid series comprised the λ-Red recombinase expressed under the control of the anhydrotetracycline (aTc)-inducible promoter P_tet_, and the Cas protein (Cas9 or Cas12a) expressed under the control of the arabinose-inducible promoter P_araB_ (Guzman et al., 1995; Cha et al., 1997) (**Figure 1A**). Because the P_araB_ promoter is repressed in presence of high glucose concentration (Guzman et al., 1995), glucose was used for inhibition of the expression of Cas12a. The donor plasmid series comprised the guide RNA(s) expressed constitutively via the J23119(SpeI) promoter (Liu et al., 2011), and donor DNA(s) as editing template(s) comprising a heterologous gene flanked by two 500 bp homologous arms, a left arm (LA) and a right arm (RA) (**Figure 1B**). Schematic maps of the donor plasmid p46Cpf1 with the native *FnCas12a* (*FnCpf1*) gene and the helper plasmid pTc-GLP which provides donor DNAs and crRNAs for three different sites are shown in **Figures 1A,B**, respectively. The plasmid construction process was described in **Supplementary Tables S1, S2**.

**Table 1:** Bacterial strains and plasmids used in this study.

Strains or plasmids	Characteristics	Source
**Strains**		
*E. coli* MG1655	F- *lambda*- *rph-1*	CGSC 6300
*H. bluephagenesis* TD01	*H. bluephagenesis* wild type	Cai et al., 2011
*E. coli* MG1655AX01	MG1655 Δ*torS*::*p103-hem1*	This study
*E. coli* MG1655AX02	MG1655 Δ*lacZ*::*T7 RNAP*, Δ*torS*::*pT7-hem1*	This study
*E. coli* MG1655AX03	MG1655 with the plasmid pLTT05	This study
**Plasmids**		
pcrRNA-P	crRNA-*pyrF*, pUC origin, *kan*	This study
p46Cas9	Expressing λ-Red and SpCas9	This study
p46Cpf1	Expressing λ-Red and wild-type FnCas12a (FnCpf1)	This study
p46Cpf1-OP1	Expressing λ-Red and codon-optimized FnCas12a (type 1)	This study
p46Cpf1-OP2	Expressing λ-Red and another codon-optimized FnCas12a (type 2)	This study (Addgene #98592)
pTs-P	sgRNA-*pyrF*, Δ*pyrF*::*gfp*	This study
pTs-PL	sgRNA-*lacZ*, Δ*lacZ*::*aadA* sgRNA-*pyrF*, Δ*pyrF*::*gfp*	This study
pTs-GLP	sgRNA-*galK*,Δ*galK*::*rfp* sgRNA-*lacZ*,Δ*lacZ*::*aadA* sgRNA-*pyrF*, Δ*pyrF*::*gfp*	This study
pTc-P	crRNA-*pyrF*, Δ*pyrF*::*gfp*	This study
pTc-P-50bp	crRNA-*pyrF*, Δ*pyrF*::*gfp* with 50 bp homology arms	This study
pTc-P-100bp	crRNA-*pyrF*, Δ*pyrF*::*gfp* with 100 bp homology arms	This study
pTc-G	crRNA-*galK*, Δ*galK*::*rfp*	This study
pTc-G2	crRNA-*galK2*, Δ*galK*::*rfp*	This study
pTc-A	crRNA-*araD*, Δ*araD*::*rpsl*	This study
pTc-A2	crRNA-*araD2*, Δ*araD*::*rpsl*	This study
pTc-M	crRNA- *mutS*, Δ*mutS*::*tcr*	This study
pTc-L	crRNA-*lacZ*, Δ*lacZ*::*aadA*	This study
pTc-GL	crRNA-*galK*, Δ*galK*::*rfp* crRNA-*lacZ*, Δ*lacZ*::*aadA*	This study
pTc-GP	crRNA-*galK*, Δ*galK*::*rfp* crRNA-*pyrF*, Δ*pyrF*::*gfp*	This study
pTc-PL	crRNA-*lacZ*, Δ*lacZ*::*aadA* crRNA-*pyrF*, Δ*pyrF*::*gfp*	This study
pTc-GLP	crRNA-*galK*,Δ*galK*::*rfp* crRNA-*lacZ*,Δ*lacZ*::*aadA* crRNA-*pyrF*, Δ*pyrF*::*gfp*	This study
pTc-arrayLP	crRNA-*lacZ-pyrF*, Δ*lacZ*::*aadA*Δ*pyrF*::*gfp*	This study
pTc-arrayPL	crRNA-*pyrF*-*lacZ*, Δ*lacZ*::*aadA*Δ*pyrF*::*gfp*	This study
pTc-torS-p103-hem1	crRNA-*torS*, Δ*torS*::*p103-hem1*	This study
pTc-lacZ-T7RNAP-torS-pT7-hem1	crRNA-*lacZ*, Δ*lacZ*::*T7 RNAP* crRNA-*torS*, Δ*torS*::*pT7-hem1*	This study
pLTT05	Expressing T7 RNA polymerase and ALA synthase (*hem1*)	Li et al., 2016
pTD-Cas12a	Expressing codon-optimized FnCas12a (type 2)	This study
pTtd-prpC	crRNA-*prpC*, Δ*prpC*	This study
pTtd-prpC-PM	crRNA-*prpC, prpC* with point mutation	This study

*kan, kanamycin resistance gene; *rfp*, red fluorescent protein gene; *aadA*, spectinomycin resistance gene; *gfp*, green fluorescent protein gene; *rpsl*, streptomycin resistance gene; *tcr*, tetracycline resistance gene; *hem1*, mitochondrial 5-aminolevulinic acid synthase gene. sgRNA-*pyrF*, sgRNA targeting the *pyrF* locus; sgRNA-*galK*, sgRNA targeting the *galK* locus; sgRNA-*lacZ*, sgRNA targeting the *lacZ* locus; crRNA-*pyrF*, crRNA targeting the *pyrF* locus; crRNA-*galK* and crRNA-*galK2*, crRNA targeting the *galK* locus; crRNA-*araD* and crRNA-*araD2*, crRNAs targeting the *araD* locus; crRNA-*mutS*, crRNA targeting the *mutS* locus; crRNA-*lacZ*, crRNA targeting the *lacZ* locus; crRNA-*torS*, crRNA targeting the *torS* locus; crRNA-*prpC*, crRNA targeting the *prpC* locus; crRNA-*lacZ-pyrF* and crRNA-*pyrF*-*lacZ*, crRNAs in a CRISPR array targeting the *galK* locus and the *lacZ* locus. Δ*pyrF*::*gfp*, editing template with two flanking 500 bp sequences homologous to the *pyrF* locus with a *gfp* insertion; Δ*galK*::*rfp*, editing template with two flanking 500 bp sequences homologous to the *galK* locus with an *rfp* insertion; Δ*araD*::*rpsl*, editing template with two flanking 500 bp sequences homologous to the *araD* locus with an *rpsl* insertion. Δ*lacZ*::*aadA*, editing template with two flanking 500 bp sequences homologous to the *lacZ* locus with an *aadA* insertion; Δ*mutS*::*tcr*, editing template with two flanking 500 bp sequences homologous to the *mutS* locus with an *tcr* insertion; Δ*lacZ*::*T7 RNAP*, editing template with two flanking 500 bp sequences homologous to the *lacZ* locus with a T7 RNA polymerase gene insertion; Δ*prpC*, editing template with two flanking 500 bp sequences homologous to the *prpC* locus. *p103-hem1*, *hem1* controlled by the 103 promoter; *pT7-hem1, hem1* controlled by the T7 promoter*.

**FIGURE 2:**
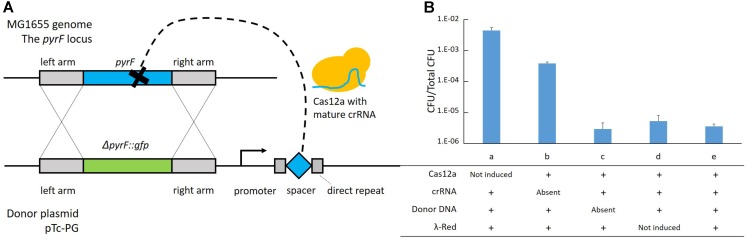
Effects of different components in the two-plasmid system on inducible cell killing. **(A)** Diagram of the events during genome editing. **(B)** Effects of Cas12a, targeting crRNA, donor DNA and λ-Red in the two-plasmid system. The data represent the means ± standard deviations from three measurements for each experiment.

In the two-plasmid system, effects of different components on inducible cell killing were tested (**Figure 2**). Cas12a processes the transcript from the donor plasmid to generate mature crRNAs (Fonfara et al., 2016). Guided by the crRNA, Cas12a finds the genomic target and induces a double-strand break (Zetsche et al., 2015) (**Figure 2A**). Recombination occurs between the genomic target and the donor DNA mediated by λ-Red (Sharan et al., 2009). We introduced intact or deficient donor plasmids into bacteria harboring the helper plasmid p46Cpf1 by electroporation. The total colony number of forming units (CFUs) refers to the number of competent cells prepared for each electroporation. The rate of CFU to total CFU indicated the survival chance of cells with intact or deficient two-plasmid systems. It was shown that Cas12a and crRNA were necessary for inducible cell killing (**Figure 2B**, cases a, b, and e). Furthermore, donor DNA has an important impact on recovery from the double-strand break caused by the CRISPR-Cas12a as the editing template (**Figure 2B**, cases c and e). Although λ-Red was considered to accelerate recombination, more cells recovered from cleavage by Cas12a when λ-Red was not induced (**Figure 2B**, cases d and e), which was probably due to a reduction of metabolic stress. It was confirmed that the inhibition of Cas12a expression by the addition of glucose prevented cell death caused by the double-strand break. This phenomenon guaranteed the coexistence of the helper plasmid with the *Cas12a* gene and the donor plasmid with the crRNA. After the necessity of the different components of the two-plasmid system was confirmed, several parameters were adjusted to achieve a high integration efficiency. Also, the λ-Red requirement was evaluated in the next section.

### Optimization of the Two-Plasmid System

We proposed to introduce the two plasmids into *E. coli* MG1655 sequentially, and incubate the obtained colonies without induction. After a certain number of cells had amplified, λ-Red and Cas12a were induced to accelerate recombination and selection. Cultures were spread on plates with L-arabinose to induce the expression of Cas12a consistently. We selected 12 colonies from each plate for PCR genotyping (**Supplementary Figure S1**). The integration efficiency was defined as the rate of the number of modified colonies to the total number of colonies that had been genotyped. A general outline of this method is shown in **Figure 1C**.

We tested the recombination system by introducing a gene at a single locus in the *E. coli* genome. To avoid affecting the growth of the modified cells, five sites with non-essential genes (*araD, galK, lacZ, mutS*, and *pyrF*) were selected for the integration of heterologous genes, and crRNAs targeting these genomic sites (**Supplementary Table S3**) were designed.

**FIGURE 3:**
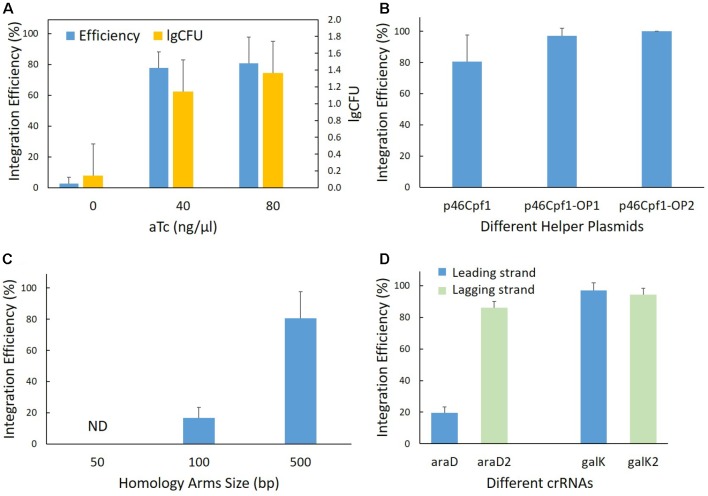
Parameters affecting recombination efficiency. **(A)** Effect of the λ-Red machinery. Genomic integration was performed at the *pyrF* site in both the λ-Red induced (aTc added) and non-induced (aTc not added) backgrounds. The helper plasmid p46Cpf1 and the donor plasmid pTc-PG were used. CFU stands for colony number of forming units per microliter of culture. **(B)** Effect of codon optimization of the *Cas12a* gene. Genomic integration was performed at the *pyrF* site using different helper plasmids - p46Cpf1 with the original *Cas12a* (*Cpf1*) gene, p46Cpf1-OP1 and p46Cpf1-OP2 with codon optimized *Cas12a* genes. The donor plasmid pTc-PG was used. **(C)** Effect of homology arm length on integration efficiency. Genomic integration was performed at the *pyrF* site with different donor plasmids, pTc-P-50bp providing the donor template with 50-bp homology arms, pTc-P-100bp with 100-bp homology arms and pTc-P with 500-bp homology arms. The helper plasmid p46Cpf1 was used. Modified colonies were not detected when using 50 bp homology arms. ND, not detected. **(D)** Relationship between integration efficiency and different crRNAs. The crRNAs araD and galK target the leading strand in the *E. coli* genome, while the crRNAs araD2 and galK2 target the lagging strand. The helper plasmid p46Cpf1 was used. The data represent the averages of three independent experiments.

Firstly, it was reported that the assistance of λ-Red was necessary in the genome editing technologies based on CRISPR-Cas9 (Jiang et al., 2015; Pyne et al., 2015; Bassalo et al., 2016), and the λ-Red requirement was also evaluated for this strategy. In the helper plasmid, the λ-Red proteins are expressed under the control of an aTc-inducible promoter, P_tet_. Therefore, it was possible to regulate λ-Red production using the inducing agent – aTc. The helper plasmid p46Cpf1 and the donor plasmid pTc-P were used to edit the *pyrF* locus in both the induced and non-induced backgrounds. While the two-plasmid system worked in the induced backgrounds at two different concentrations, almost no modified cells were observed in the non-induced backgrounds (**Figure 3A**), underlining the necessity of λ-Red for efficient recombination. Although, from the experimental results, an increase of the aTc concentration from 40 to 80 ng/μl did not have a significant effect on integration efficiency or the amount of surviving cells, we chose the aTc concentration of 80 ng/μl in subsequent experiments to ensure the adequate supply of λ-Red recombinases.

Secondly, we aimed to improve the expression of Cas12a for effective selection. One option was to use a strong promoter or ribosome binding site (RBS) to augment the amount of messenger RNA (mRNA). The *Cas12a* gene, however, is very large (∼4 kb), which causes a great burden for the cells when it is transcribed at high levels. Thus, codon optimization of the *Cas12a* gene was conducted to improve the production of mature Cas12a while keeping the mRNA content at the original level. To this end, two different codon optimization strategies were employed. The *Cas12a* (*Cpf1*) gene in p46Cpf1-OP1 was optimized using OPTIMIZER (Puigbo et al., 2007), whereas in p46Cpf1-OP2, another tool, JCat (Grote et al., 2005) was utilized (**Supplementary Table S3**). The same donor plasmid, Tc-P (**Table 1**), was harnessed for recombination at the *pyrF* site, while three different donor plasmids were tested that supplied the λ-Red proteins and Cas12a in different quantities. The results are shown in **Figure 3B** and **Supplementary Figure S2**. Although the results were not statistically significant to prove a more efficient recombination or selection assisted by p46Cpf1-OP1 or p46Cpf1-OP2, an increased average integration efficiency was observed after the *Cas12* gene was codon optimized. We presumed that production enhancement of mature Cas12a had a positive impact on the recombination assays.

Thirdly, we tested different lengths of the homology arms. At the *pyrF* site, recombination templates with 50 bp (pTc-P-50bp), 100 bp (pTc-P-100bp) and 500 bp (pTc-P) homology arms were used. As a result, 50 bp homology arms were insufficient for recombination, and integration efficiency increased dramatically when the homology arms were elongated from 100 bp to 500 bp (**Figure 3C**).

Finally, the effect of different crRNAs was explored. We designed two crRNAs at each site for *araD* and *galK*, respectively. The plasmids pTc-G and pTc-A expressed crRNAs targeting the leading strand in the *E. coli* genome, while those expressed by pTc-G2 and pTc-A2 targeted the lagging strand. It was interesting that the two crRNAs targeting the *araD* site had markedly different appearance. At the *galK* site, however, the results of using different crRNAs were similar (**Figure 3D**). There was not enough evidence to draw a conclusion on effects of crRNAs targeting different strands. Nevertheless, it is inferred better to design at least two crRNAs for a locus in order to ensure a functional one.

### Performing Gene Insertion at a Single Locus

**FIGURE 4:**
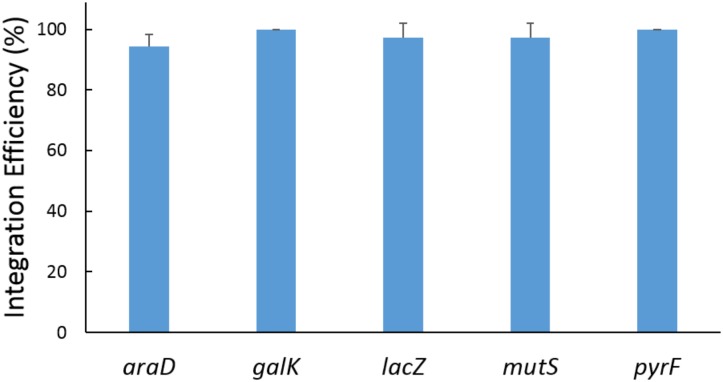
Results of recombination at a single locus. The five loci were edited in five independent experiments. In each experiment, a single locus among the five loci was edited. The donor plasmids pTc-A2, pTc-G, pTc-L, pTc-M, and pTc-P provided donor DNA and crRNAs for the *araD, galK, lacZ, mutS*, and *pyrF* site, respectively. The helper plasmid p46Cpf1-OP2 was used. The data represent the averages of three independent experiments.

The 5 donor plasmids (Tc-A2, Tc-G, Tc-L, Tc-M, and Tc-P), providing a series of paired crRNAs and donor DNAs, were combined in the recombination assays with the helper plasmid p46Cpf1-OP2. In each round of experiment, 12 colonies were genotyped by PCR, and one of the resulting modified clones was verified by DNA sequencing to confirm the integration of the heterologous gene (**Supplementary Figure S3**). As shown in **Figure 4**, the integration efficiency was almost 100% at each site. The five loci (*araD, galK, lacZ, mutS*, and *pyrF*) are scattered in different places on the chromosome, indicating that the developed method is applicable on the whole genome.

### Simultaneous Recombination at Multiple Loci

We next investigated if this method can be used to simultaneously insert genes at multiple loci of the *E. coli* chromosome.

**FIGURE 5:**
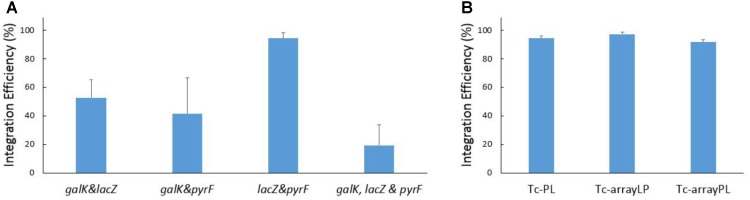
Results of simultaneous recombination at multiple sites. **(A)** Efficiency of simultaneous integration at two and three sites. The helper plasmid p46Cpf1-OP2 was used. Different combination of two cRNAs against the three selected target sites (*galK, lacZ*, and *pyrF*) were used in the recombination experiment. The donor plasmids pTc-GL was used for recombination at the *galK* and *lacZ* sites; pTc-GP, *galK* and *pyrF* sites; pTc-PL, *lacZ*, and *pyrF* sites. And the donor plasmid pTc-GLP was used for simultaneous recombination at all the three sites (*galK, lacZ*, and *pyrF*). **(B)** Effect of different ways of supplying the crRNAs. The donor plasmid pTc-PL expresses two crRNAs separately, while the donor plasmids pTc-arrayLP and pTc-arrayPL express crRNAs arranged in a CRISPR array. The helper plasmid p46Cpf1-OP2 was used. The data represent the averages of three independent experiments.

Firstly, we attempted to insert genes at two sites simultaneously. Different combination of two cRNAs against the three selected target sites (*galK, lacZ*, and *pyrF*) were tested. As shown in **Figure 5A**, the introduction of both heterologous genes was confirmed in more than 40% of the colonies when p46Cpf1-OP2 was utilized as the helper plasmid.

Subsequently, simultaneous insertion in all the three loci was conducted using the donor plasmid pTc-GLP and the helper plasmid p46Cpf1-OP2 (**Figure 5A** and **Supplementary Figure S4**). As we had anticipated, the integration efficiency decreased dramatically with the increase in the number of simultaneous insertions. Nevertheless, simultaneous gene insertion into three sites was achieved with an acceptable efficiency. One colony with all three desired modifications appeared on average among every five colonies, meaning that researchers should be able to obtain the desired strain through limited work spent on PCR genotyping.

Furthermore, several crRNAs can be arranged in a CRISPR array that can be processed by Cas12a itself (Fonfara et al., 2016). This feature of the CRISPR-Cas12a system makes it convenient for the expression of crRNAs targeting multiple loci. In order to compare different ways of supplying crRNAs, pTc-arrayLP and pTc-arrayPL were constructed, with crRNAs expressed in a CRISPR array in different orders. By contrast, in pTc-PL the crRNAs were expressed separately. Recombination assays were conducted using these donor plasmids and the helper plasmid p46Cpf1-OP2.Importantly, supplying crRNAs in a CRISPR array did not reduce the integration efficiency (**Figure 5B**).

### Comparing the Two-Plasmid Systems Based on CRISPR-Cas12a and CRISPR-Cas9

**FIGURE 6:**
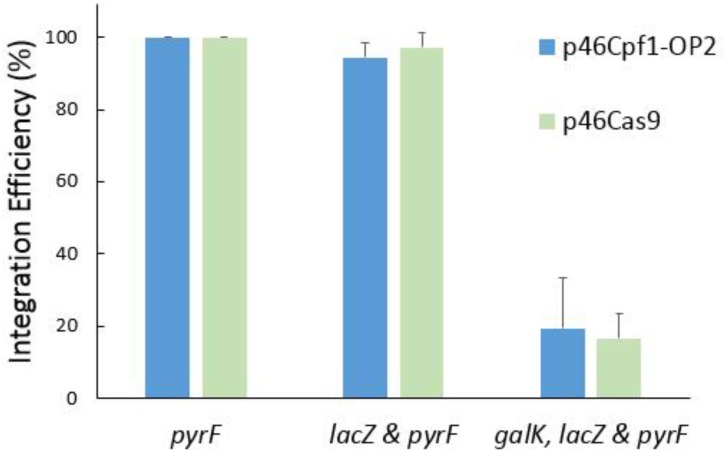
Recombination efficiency of different CRISPR systems. The helper plasmid p46Cpf1-OP2 was combined with the donor plasmids pTc-P, pTc-PL, and pTc-GLP to perform simultaneous modifications at a single locus (*pyrF*), two loci (*lacZ* and *pyrF*) and three loci (*galK, lacZ*, and *pyrF*), respectively. For recombination based on CRISPR-Cas9, p46Cas9, pTs-P, pTs-PL, and pTs-GLP were used. The data represent the averages of three independent experiments.

To explore the differences of the efficiency of Cas12a and Cas9 in recombination and counterselection, both CRISPR-Cas systems were employed to build helper plasmids and donor plasmids. Recombination at a single locus, two loci and three loci was tested. As shown in **Figure 6**, the systems based on *Cas9* and codon-optimized *Cas12a* had similar performance.

Assisted by CRISPR-Cas12a or CRISPR-Cas9, the integration efficiency at a single locus was almost 100% (**Figures 4**, **6**). However, at multiple separate loci, it might decreased significantly. For example, at the *galK* and *pyrF* loci, the integration efficiency was about 40% (**Figure 5A**) and it dropped to about 20% when performing recombination simultaneously at 3 loci (**Figure 6**). It was reported that cells escaping from Cas9 cleavage might carry a defective CRISPR system (Cui and Bikard, 2016). The similar performance of CRISPR-Cas12a and CRISPR-Cas9 in the two-plasmid system convinced us that cells could also escape from Cas12a cleavage due to the deficiency in the CRISPR system. When double-strand breaks were introduced to multiple separate loci in the chromosome at the same time, it might be difficult to repair all the breaks through homologous recombination. Less cells survived through recombination between the chromosome and the donor DNA, while more cells carried a defective CRISPR system, leading to the dramatic decrease in integration efficiency.

### Construction of Recombinant *E. coli* Strains for the Production of ALA

To demonstrate the practical application of this gene-insertion method, we constructed recombinant *E. coli* strains for the production of ALA, an industrially useful chemical. In the past, we constructed a series of strains for the production of ALA which carry the codon-optimized mitochondrial 5-aminolevulinic acid synthase (EC: 2.3.1.37, *hem1*) gene on plasmids (Li et al., 2016). Although expressing genes from high-copy-number plasmids enables high output, episomal plasmids are sometimes unstable (Ganusov and Brilkov, 2002) and require the addition of antibiotics during fermentation (Godwin and Slater, 1979), which makes chromosomal integration of metabolic pathways a promising alternative (Englaender et al., 2017). In this study, we achieved to integrate the *hem1* gene into the *E. coli* chromosome using the developed gene inserting method.

**FIGURE 7:**
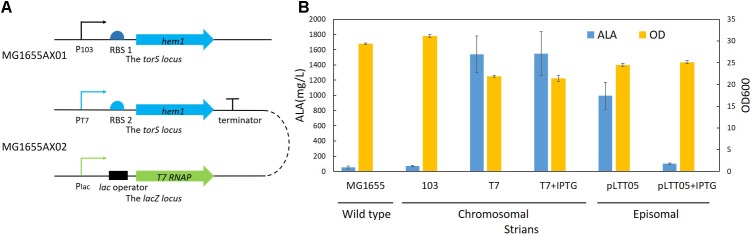
ALA production by different strains. **(A)** A schematic of the genetically modified MG1655 strains. **(B)** Results of ALA production. 103 stands for the strain MG1655AX01 which carries a chromosomally integrated *hem1* gene controlled by the 103 promoter; T7 stands for the strain MG1655AX02 which carries a chromosomally integrated *hem1* gene controlled by the T7 promoter and a chromosomally integrated *T7 RNA polymerase* gene controlled by the native lac promoter; pLTT05 stands for the strain MG1655AX03 which harbors the plasmid pLTT05. pT7 + IPTG and pLTT05 + IPTG denote that IPTG was added during the fermentation of the corresponding strains.

To obtain a high-yielding recombinant strain, we selected two promoters to control the *hem1* gene: the constitutive 103 promoter (P_103_) and the T7 promoter (P_T7_). The p103-*hem1* cassette (∼1.8 kb, **Supplementary Table S5**) was integrated into the *torS* site to construct the strain MG1655AX01 (**Table 1** and **Figure 7A**). The strain MG1655AX02 (**Table 1** and **Figure 7A**) was obtained by simultaneously integrating the *T7 RNAP* cassette (∼2.6 kb, **Supplementary Table S5**) into the *lacZ* site and the pT7-*hem1* cassette (∼2.0 kb, **Supplementary Table S5**) into the *torS* site. In MG1655AX02, *T7 RNAP* was under control of the native *lac* promoter (P_lac_), which is isopropyl-β-D-thiogalactoside (IPTG)-inducible. We introduced the plasmid pLTT05 into MG1655 to obtain the strain MG1655AX03 (**Table 1**), in which the *hem1* gene was controlled by a T7lac promotor and *T7 RNAP* was expressed constitutively. These strains were used for fermentation. The best average yield of the recombinant strain MG1655AX02 was 1.55 ± 0.29 g/L, a performance better than that of MG1655AX03 with an episomal *hem1* gene (**Figure 7B**), which was probably the result of the usage of a strong RBS. Intriguingly, MG1655AX02 had similar performance in induced and non-induced conditions while MG1655AX03 produced much less ALA when induced by IPTG. These results implied that the proteins leaking from the IPTG-inducible promoters (P_lac_ or P_T7lac_) might be enough for efficient production of ALA and that excessive expression of T7 RNA polymerase or ALA synthase was a burden for bacteria. Although we made sure that nearby promoters in the upstream of *hem1* or *T7RNAP* would not influence the protein expression by positioning them in the opposite direction, it was noteworthy that random sequences could somehow serve as active promoters. Thus, terminators should be added in the upstream of these genes to insulate them from outside influence.

### Editing the Genome of *Halomonas bluephagenesis*

Recently, a method based on CRISPR-Cas9 for editing the genome of the extremophilic *Halomonas* spp. was reported (Qin et al., 2018). Notwithstanding, CRISPR-Cas12a can be used when there are no appropriate targets for Cas9, because they recognize different PAM regions. To test the potential of CRISPR-Cas12a in editing the genome of different bacterial species, we built another two-plasmid system for genome editing in *H. bluephagenesis*, comprising the helper plasmid pTD-Cas12a and a donor plasmid, for example, pTtd-prpC (**Table 1**). Similar strategies as reported by Qin et al. (2018) were exploited to perform the deletion of *prpC* in the chromosome of *H. bluephagenesis*. pTtd-prpC was used to delete the *prpC* gene, and pTtd-prpC-PM was used to introduce a point mutation into the PAM sequence in the target of crRNA-*prpC*, generating a premature stop codon (**Supplementary Figure S5**). After genotyping of the resulting colonies (**Supplementary Figure S5**), modified cells were sequenced to confirm the mutations. Thus, it was demonstrated that the CRISPR-Cas12a system can be used for genome editing in a non-model species of bacteria, the extremophilic *H. bluephagenesis.*

## Discussion

In this study, we developed a fast and convenient genome editing method based on the CRISPR-Cas12a system, which can perform gene insertions at multiple loci simultaneously (**Table 2**). Our system is capable of editing two genes simultaneously with high efficiency (more than 40%), and three genes simultaneously with lower, but detectable efficiency (about 20%). Compared with Cas9, the smaller size of Cas12a enabled the use of smaller plasmids, and therefore allowed easier manipulation (plasmid construction, electroporation, *etc*.). The T-rich PAM of Cas12a, which differs from the G-rich PAM of Cas9, should expand the scope of target sites in the organism’s genome. Similar to published genome editing technology based on CRISPR-Cas9, our system can perform rapid gene insertion in a single recombination step, whereas other scarless genome editing methods involving I-SceI (Pósfai et al., 1999; Yu et al., 2008; Yang et al., 2014) take two steps to obtain a modified strain. The CRISPR-Cas9 technology (**Table 2**) is able to perform multiple gene deletions at one time. However, its ability to integrate multiple genes into the chromosome has not been verified (Jiang et al., 2015; Li et al., 2015). Although another genome editing technology based on CRISPR-Cas9, the no-SCAR method (Reisch and Prather, 2015), achieves multiple genomic modifications iteratively, it takes more time than our system when performing modifications on multiple loci. Using the no-SCAR method, it takes 11 days to obtain a plasmid-free strain with two mutations and 14 days for a strain with three, while we were able to construct a strain with three mutations in 8 days (**Table 2**).

**Table 2:** Comparisons of genome editing methods assisted by CRISPR-Cas system.

Comparison	Method	This study	Cas9-assisted method (Li et al., 2015)	Cas12a-assisted method (Yan et al., 2017)	NO SCAR (Reisch and Prather, 2015)
Component	*Cas* gene	Codon-optimized *FnCas12a*	*SpCas9*	*FnCas12a*	*SpCas9*
	Donor DNA	Circular (plasmids)	Linear (PCR products)	Linear (PCR products or oligonucleotides)	Linear (PCR products or oligonucleotides)
Editing efficiency	At a single locus	Nearly 100%	Nearly 100%	More than 50%	85 ∼ 100%
	At multiple loci	About 20% for 3 gene insertions	About 20% for 3 point mutations	Not tested	Not tested
Time	1 mutation	8 days	7 days	7 days	8 days
	2 mutations	8 days	13 days	13 days	11 days
	3 mutations	8 days	19 days	19 days	14 days

Several parameters were adjusted to improve the two-plasmid system, including the usage of codon-optimized *Cas12a*. Also, by arranging crRNAs separately or in a CRISPR array, different ways to supply crRNAs were explored and showed similar performance. Differences in the recombination and counterselection ability between the systems based on Cas12a and Cas9 were explored and it was proved the system based on *Cas9* performed similarly to those based on codon-optimized *Cas12a*. In addition, the loss of protospacer between direct repeat sequences in crRNA was observed, and novel strategies for supplying guide RNAs have a potential to address this limitation (Gao and Zhao, 2014; Port and Bullock, 2016; Xu et al., 2017) and to improve this genome editing method.

It was recently reported that CRISPR-Cas12a has indiscriminate single-stranded DNase activity (Chen et al., 2018). Since we used circular DNAs (sequences as part of the donor plasmid) as donor templates, such single-stranded DNase activity should have little impact on the recombination events, ensuring the success of multiplex genome engineering of up to 3 loci. We speculated that it would be necessary to avoid using linear DNAs (PCR products or oligonucleotides) as editing templates to increase the editing efficiency.

The λ-Red recombinase complex, comprising Gam, Bet and Exo, is necessary to promote recombination (Datsenko and Wanner, 2000; Jiang et al., 2015). Exo is an exonuclease which digests double-stranded DNA (dsDNA) from 5^′^ to 3^′^, generating single-stranded DNA (ssDNA). Bet is an ssDNA binding protein (Murphy, 1998; Zhang et al., 1998). The λ-Red component Gam is able to block the RecBCD complex to protect the DNA template from digestion (Murphy, 1991; Marsiæ et al., 1993). It is believed that the generated ssDNA acts as the template during recombination (Stahl et al., 1997). However, instead of linear DNAs, we used circular DNAs (donor plasmids) as editing templates, which makes it challenging to process the circular templates into ssDNA. What’s more, CRISPR-Cas12a has indiscriminate single-stranded DNase activity. Considering these features of the system, we deduced that an altered recombination mechanism must mediate the functioning of our two-plasmid system and that the double-stand breaks (DSBs) caused by the CRISPR system had a vital role of triggering the recombination events other than selection. Previous research based on the CRISPR-Cas9 system showed that when using 50-bp homology arms and PCR products as templates, the editing efficiency of 1 kb insertions is nearly 50% (Li et al., 2015). However, when using 50-bp homology arms and circular templates in our system, no modified cells were detected (**Figure 3C**). The hypothesis of different recombination mechanisms is therefore a reasonable explanation for this phenomenon.

To demonstrate the practical application of this multiplex gene-insertion method, we constructed a recombinant *E. coli* producing an industrially useful chemical, ALA, at high yield. Moreover, a similar two-plasmid system was built to edit the genome of the extremophile *H. bluephagenesis* which indicates the promising potential of the CRISPR-Cas12a system in assisting genome editing in different bacterial species, including non-model organisms.

In **Supplementary Table S6**, we summarized the above-mentioned recombination assays. When developing the two-plasmid system, the culture of each assay was spread onto two plates – one with L-arabinose to keep the expression of Cas protein and the other with glucose to inhibit the expression. The rate of colony number on the L-arabinose plate to that on the glucose plate (CFU_arabinose_/CFU_glucose_) was between 0.1 and 1.0 (**Supplementary Table S6**). However, when coupling the recombination with electroporation, the survival chance of cells with intact two-plasmid systems was much lower (**Figure 2B**, column e). Introducing the donor plasmid into cells beforehand was then a considerable approach to accelerate genome editing.

In summary, we demonstrated that the developed method enables rapid and efficient gene insertions at up to three genomic loci simultaneously in a “markerless” and “scarless” manner. We obtained a plasmid-free strain with three heterologous genes integrated into multiple loci of the chromosome in just 8 days. This method should therefore expedite multiplex genome editing in *E. coli* and benefit further engineering and synthetic biology studies.

## Materials and Methods

### Bacterial Strains and Plasmids

All strains and plasmids used in this study are listed in **Table 1** and **Supplementary Table S1**. *E. coli* strain Trans1-T1 (TransGen Biotech Co., Ltd, China) was used as the host strain for plasmid construction, and *E. coli* strain MG1655 (CGSC 6300) was used as targets for genome engineering. Primers used for plasmid construction are listed in **Supplementary Table S2**. Sequences encoding the synthetic guide RNA (sgRNA) (Jinek et al., 2012), crRNAs, the *spCas9* gene, the wild-type *FnCas12a* gene (Zetsche et al., 2015) and the two *FnCas12a* gene variants codon-optimized were synthesized by Qinglan Biotech (Suzhou, China) (**Supplementary Table S3**). The selected targets for the CRISPR-Cas system in the *E. coli* genome are listed in **Supplementary Table S3**. After DNA amplification, restriction enzyme digestion, ligation and other standard molecular cloning standard procedures were performed to construct the plasmids. We used DNA purification and plasmid isolation kits from Biomed (Beijing, China). The Q5 polymerase was purchased from New England Biolabs (Beijing, China). The restriction enzymes and ligation kits were purchased from Thermo Fisher Scientific (China).

### Testing Effects of Components in the Two-Plasmid System

MG1655 harboring p46Cpf1 was incubated with the corresponding inducer/inhibitor (aTc and L-arabinose/glucose) and then processed into competent cells (Sharan et al., 2009). We spread 50 μL of electrocompetent cells on LB plates to calculate total CFU. Next, 100 ng of different plasmids was introduced into 50 μL of electrocompetent cells. After 1-h incubation at 30°C and 200 rpm, the cells were spread on LB plates with chloramphenicol, kanamycin and L-arabinose/glucose. CFUs were calculated after 1-day incubation at 30°C. The ratio of transformed CFU to total CFU is shown in **Figure 2B**. In case a, Cas12a (Cpf1) was not induced due to absence of L-arabinose and presence of glucose; in case b, c, and e, both Cas12a and λ-Red were induced due to presence of aTc and L-arabinose; in case d, λ-Red was not induced due to absence of aTc. In case a, d, and e, the helper plasmid pTc-P was used to provide the crRNA and the donor DNA; in case b, the helper plasmid pTs-P was used to provide the donor DNA while the crRNA in CRISP-Cas12a system was absent; in case c, the plasmid pcrRNA-P was used to provide the crRNA in CRISPR-Cpf1 system while the donor DNA was absent.

### Recombination Assisted by λ-Red and the CRISPR-Cas System

A general outline of this method is shown in **Figure 1C**.

Day 1: Grow MG1655 in LB at 37°C overnight. Next morning, add 0.2 mL of the culture to 20 ml of LB in a 100-mL shake flask and grow at 37°C to an OD_600_ of 0.6–0.8. Centrifuge the culture at 4,000 *g* for 5 min at 4°C. Wash the cell pellet with 20 mL of ice-cold water once and then resuspended in 1 mL of ice cold water and transfer to a 1.5-mL tube. Centrifuge at 4,000 *g* for 2 min at 4°C. Wash the cells two more times with 1 mL of ice-cold 10% (v/v) glycerol. Resuspend the cell pellet in ice-cold 10% (v/v) glycerol in a final volume of 1 mL. Mix about 100 ng of the helper plasmid with 50 μL of electrocompetent cells then transfer into a 2-mm Gene Pulser cuvette (Bio-Rad, United States). Introduce the helper plasmid into the cells by electroporation at 1.8 kV. After electroporation, add 1 ml of LB and transfer the cells to a 1.5-mL tube. Incubate the cells at 30°C for 1 h. Plate 100 μL of the cell suspension onto an LB plate with 12.5 μg/mL chloramphenicol and incubate at 30°C 20–22 h. (Tips: Chemical transformation is feasible as well.)

Day 2: Pick an individual colony and grow in LB with 12.5 μg/mL chloramphenicol at 30°C and 200 rpm overnight.

Day 3: Add 0.2 mL of the culture of the intermediate strain to 20 mL of LB with 12.5 μg/mL chloramphenicol and 20 g/mL glucose (to inhibit transcription from the ParaB promoter) in a 100-mL shake flask and grow at 30°C to an OD_600_ of 0.6–0.8. Process the intermediate strain into competent cells (same procedure as above). Introduce about 100 ng of the donor plasmid into the cells via electroporation in a 2-mm Gene Pulser cuvette (Bio-Rad) at 1.8 kV, after which add 1 mL LB liquid medium with 20 mg/mL glucose to the electroporated cells. Regenerate at 30°C for 1 h and plate 100 μL of the cell suspension onto an LB plate with 12.5 μg/mL chloramphenicol, 50 μg/mL kanamycin and 20 mg/mL glucose and incubate at 30°C for 20–22 h. (Tips: Chemical transformation is feasible as well.)

Day 4: Pick an individual colony and grow in LB with 12.5 μg/mL chloramphenicol, 50 μg/mL kanamycin and 20 mg/mL glucose at 30°C and 200 rpm overnight.

Day 5: Add 10 μL of the resulting overnight seed culture to a culture tube containing 1 mL LB medium with 50 μg/mL kanamycin and 12.5 μg/mL chloramphenicol. Cultivate at 30°C and 200 rpm for 6 h, then add 5 mg/mL L-arabinose and 80 ng/mL aTc to the mixture. After a further 2 h of incubation, spread a serial dilution of the harvested culture (100, 10, and 1 μL as well as 0.1 μL of the culture) on LB plates supplemented with 5 mg/mL L-arabinose, 50 μg/mL kanamycin and 12.5 μg/mL chloramphenicol. (Tips: It is recommended that inducers be added when the turbidity of the culture is visible to the naked eye. In case the bacteria grow slowly, incubate the culture over night before spreading it on plates.)

Day 6: After overnight incubation at 30°C, confirm the corresponding strains by PCR genotyping.

### PCR Genotyping

PCR genotyping was carried out to distinguish wild-type cells from those with the desired modifications. The corresponding primers are listed in **Supplementary Table S4**. Hotstart Taq polymerase, which does not possess the 3^′^ to 5^′^ exonuclease activity was purchased from TransGen Biotech Co., Ltd., China. Gene insertions were confirmed by PCR amplification using a forward primer targeting a sequence upstream of the genomic locus (at a distance of more than 500 bp from the transcription-initiation site) and a reverse primer binding the inserted gene. In order to detect wild-type cells, one primer of the pair was designed to bind the genomic sequence which should disappear after recombination. Twelve colonies were genotyped in each round of experiment. The schematic diagrams were provided in **Supplementary Figure S1**. In some cases, both genotypes were detected in a single colony (**Supplementary Figures S2B,C**, arrows), which means that such a colony was composed of both wild-type and modified cells. A mutant colony was confirmed as successfully modified only if no wild-type cells were detected. The integration efficiency stands for the ratio of successfully modified colony number to total colony number in the PCR genotyping test.

### Plasmid Curing

The confirmed colonies were picked and grown in 2 mL of LB at 42°C and 200 rpm overnight. Next morning, the culture was diluted and spread onto LB plates. After 12 h’ incubation at 37°C, 24 resulting colonies were picked and each of them was inoculated onto three distinct plates – an LB plate supplemented with 50 μg/mL kanamycin, an LB plate supplemented with 12.5 μg/mL chloramphenicol and a further LB plate without antibiotics. The colonies sensitive to both antibiotics were confirmed as plasmid-free.

### Construction of the Recombinant *E. coli* Strain and Production of ALA

The plasmids p46Cpf1-OP2 and pTc-torS-p103-hem1 were used to construct the recombinant *E. coli* MG1655AX01 with the *hem1* gene controlled by P_103_ inserted into the *torS* site. The plasmids p46Cpf1-OP2 and pTc-lacZ-T7RNAP-torS-pT7-hem1 were used to construct the recombinant *E. coli* MG1655AX02 with the *hem1* gene controlled by P_T7_ inserted into the *torS* site and the *T7 RNA polymerase* gene into the *lacZ* site. At the *torS* site, the primer pair AX069/AX070 (**Supplementary Table S4**) was used in genotyping PCR to verify the modified cells, while the primer pair AX067/AX068 (**Supplementary Table S4**) was used to verify wild-type cells.

The tested strains were incubated in 20 mL of LB medium at 37°C and 200 rpm overnight to form seed cultures. 1 mL of the seed culture was inoculated into a 50-mL shake flask containing 20 mL of pH-adjusted LB medium (10 g/L tryptone, 5 g/L yeast extract, 83.4 mM K_2_HPO_4_∙3H_2_O, 216.6 mM KH_2_PO_4_, 3.0 g/L glycine and 6.0 g/L succinic acid). Where appropriate, 2 mM isopropyl-β-D-thiogalactoside (IPTG) was added at the beginning of the fermentation. The fermentation was conducted at 30°C and 200 rpm. After 18 h of incubation, an additional 10.0 g/L glucose, as well as 5.0 g/L glycine and 10.0 g/L succinic acid were added. The supernatant containing ALA was harvested after 48 h of fermentation. The experiments were carried out in triplicate and the ALA produced by the culture was quantified using a classical method (Mauzerall and Granick, 1956).

### Editing the Genome of *Halomonas bluephagenesis*

The procedures for genome editing of *H. bluephagenesis* using the method based on CRISPR-Cas12a were developed according to that based on CRIPSR-Cas9 (Qin et al., 2018). Generally, the helper plasmid pTD-Cas12a was introduced into *H. bluephagenesis* TD01 by conjugation, followed by the donor plasmid pTtd-prpC or pTtd-prpC-PM (**Table 1**). After at least 36 h of incubation, the resulting colonies were genotyped by PCR.

## Author Contributions

XA, YY, YG, and QW designed the experiments. XA, YY, TL, YG, T-TY, XD, and Z-TZ performed the experiments. G-QC contributed intellectual input. XA interpreted the results and wrote the paper.

## Conflict of Interest Statement

The authors declare that the research was conducted in the absence of any commercial or financial relationships that could be construed as a potential conflict of interest.
