# Supplementary material for: A Multiplex Genome Editing Method for Escherichia coli Based on CRISPR-Cas12a
Source: Front Microbiol. 2018 Oct 9;9:2307. doi: 10.3389/fmicb.2018.02307 (PMC6189296; doi:10.3389/fmicb.2018.02307)
Supplement: Supplementary file 1 [file Data_Sheet_1.docx]

**A multiplex genome editing method for *Escherichia coli* based on CRISPR-Cas12a**

**Xiang Ao^1,2,3,4+^, Yi Yao^1,3,4+^, Tian Li^5^, Ting-Ting Yang^3^, Xu Dong^3^, Ze-Tong Zheng^3^, Guo-Qiang Chen^1,2,3,4^, Qiong Wu^1,3,4^* and Yingying Guo****^6^***

^1^MOE Key Laboratory of Bioinformatics, Center for Synthetic and Systems Biology, Tsinghua University, Beijing, China, ^2^Tsinghua-Peking Center for Life Sciences, Tsinghua University, Beijing, China, ^3^School of Life Sciences, Tsinghua University, Beijing, China, ^4^Center for Synthetic & Systems Biology, Tsinghua University, Beijing, China, ^5^China National Center for Biotechnology Development, Beijing, China, ^6^State Key Laboratory of Environmental Chemistry and Ecotoxicology, Research Center for Eco-Environmental Sciences, Chinese Academy of Sciences , Beijing, China. ^+^These authors contributed equally to this work.

*** Correspondence:**

Yingying Guo

guoyingying119@126.com

Qiong Wu

[wuqiong@mail.tsinghua.edu.cn](mailto:wuqiong@mail.tsinghua.edu.cn)

**Supplementary information**

**Supplemental Methods**

**Plasmids and construction.**

| Plasmid | Characteristics | Source or reference |
| --- | --- | --- |
| pKD46 | *repA101*(Ts) *bla araC* *P_araB_-Red* | 1 |
| p46C | *repA101*(Ts) *cat tetR* *P_tet_-Red* | This study |
| pCloD-CmR | CloDF13 ori, *cat*, P_araB_ | This study |
| pCloD-Cas9 | Expressing *SpCas9* | This study |
| pCloD-Cas12a | Expressing wild-type *FnCas12a* | This study |
| pCloD-Cas12a-OP1 | Expressing codon optimized *FnCas12a* | This study |
| pCloD-Cas12a-OP2 | Expressing another codon optimized *FnCas12a* | This study |

**Table S1. General plasmids used in this study.** *bla*, ampicillin resistance gene; *P_araB_-Red*, the Red recombination genes with an arabinose-inducible promoter; *P_tet_-Red*, the Red recombination genes with promoter P_tet_.

CloDF13 ori was amplified using AX001/AX002 and digested with *Sal*I/*Sac*I. *Cat* was amplified using AX003/AX004 and digested with *Kpn*I/*Sac*I. The lambda t0 terminator was amplified using AX005/AX006. The *araC*-P_araB_ sequence from pKD46 was amplified using AX007/AX008. Overlap elongation of the lambda t0 terminator and *araC*-P_araB_ sequence was conducted with AX005/AX008 and the product was digested with KpnI/SalI. pCloD-CmR was constructed by ligating the above three fragments. pCloD-Cas12a, pCloD-Cas12a-OP1 and pCloD-Cas12a-OP2 were constructed by ligation of pCloD-CmR and synthetic Cas12a sequences digested with *Apal*I/*Sph*I. pCloD-Cas9 was constructed by ligation of pCloD-CmR and a synthetic Cas9 sequence digested with *Apal*I/*Sal*I. *Cat* was amplified using AX009/AX010 and digested with *Eco*31I/*Xma*JI. The *tetR*-Ptet sequence was amplified using AX011/AX012 and digested with *Eco*31I. The lambda Red sequence from pKD46^1^ was amplified using AX013/AX014 and digested with *Xma*JI/*Xho*I. p46C was constructed by ligation of the above three fragments. P46C was digested with *Apal*I/*Xma*JI. The variant Cas protein genes were amplified using AX015 and AX016, using pCloD-Cas9, pCloD-Cas12a, pCloD-Cas12a-OP1 and pCloD-Cas12a-OP2 as templates, respectively, and digested with *Eco*31I/*Xma*JI. The resulting fragments were then ligated into a digested p46C backbone, yielding p46Cas9, p46Cpf1, p46Cpf1-OP1 and p46Cpf1-OP2, respectively. The *kan*-pBR322 ori was amplified using AX017/AX018 and digested with *Xba*I/*Xho*I. AX019 and AX036 were used to amplify homologous arms from the MG1655 genome and heterologous genes for insertion. The right and left homologous arms, genes for insertion and the *kan*-pUC ori sequence were ligated after digestion to form intermediate plasmids. The intermediate plasmids were amplified using AX037/AX038, digested with *Mfe*I/*Sal*I, and ligated to fragments obtained from synthetic guide RNA sequences via digestion with *Mfe*I/*Sal*I (Table S2) to form the series of donor plasmids. Fragment 1 from Tc-G was digested with *Sal*I/*Xba*I. Fragment 2 and fragment 3 were amplified rom pTc-L and pTc-P, respectively, using AX040/AX041, followed by digestion with *Eco*31I. Fragment 4 was amplified using AX031/AX039 from pTc-P and digested with *Eco*31I/*Xba*I. Ligation of fragments 1 and 2 yielded pTc-GL; 1 and 3 yielded pTc-GP; and 2 and 4 yielded pTc-PL. The fragment amplified from pTc-PL using AX040/AX041 was digested with *Eco*31I and then ligated to fragment 1 to form pTc-GLP. pTs-P, pTs-PL and pTs-GLP were constructed in a similar way. AX055/AX056 and AX057/AX058 were used to amplify donor DNAs from pTc-P. The resulting fragments were digested with *Sal*I/*Xba*I and ligated to the crRNA fragment to yield pTc-P-50 and pTc-P100, respectively. Fragment 5 harboring the porin promoter was amplified from pRE112-pMB1^2^ using AX069/AX070.

**References**

1 Datsenko, K. A. & Wanner, B. L. One-step inactivation of chromosomal genes in Escherichia coli K-12 using PCR products. *Proceedings of the National Academy of Sciences* **97**, 6640-6645 (2000).

2 Fu, X.-Z. *et al.* Development of Halomonas TD01 as a host for open production of chemicals. *Metabolic engineering* **23**, 78-91 (2014).

**Supplemental Tables and Figures**

| No. | Name | Sequence |
| --- | --- | --- |
| AX001 | colDF | atatagtcgacatatagcggccgcactgcggcgggcgctgcggac |
| AX002 | colDR | atatcgagctcgctttacatttgaagagataaat |
| AX003 | CMF | tgtaaagcgagctcgatatcaaattacgccccgccctgcc |
| AX004 | CmR | gagtcggtaccgatatctggcgaaaatgagac |
| AX005 | ara1 | tatcggtaccgactcctgttgatagatcca |
| AX006 | ara2 | gttgtcataataaatcgatgattctcaccaataaaaaacg |
| AX007 | ara3 | gcgttttttattggtgagaatcatcgatttattatgacaac |
| AX008 | ara4 | atatagcatgctatatgtgcacatggagaaacagtagagagt |
| AX009 | cat-f-Eco31I | atataggtctcttcgactgatcggcacgtaagaggttccaa |
| AX010 | CmR-r-Alw44I-XmaJI | atatacctaggatatagtgcacaagcgagctcgatatcaaat |
| AX011 | tetR-Cr-XhoI-Eco31I | atataggtctcctcgagcatgcgacgtcttaagaccca |
| AX012 | Ptet-RBS-r-Eco31I-GATC | atataggtctcagatccctttctcctctttagatcttttga |
| AX013 | 101-r-XmaJI | atatacctaggcccttaacgtgagttttcgtt |
| AX014 | 101-f-XhoI | aaaggctcgagaaaaaatggatattaatactgaaactgagatcaagcaaa |
| AX015 | lamda t0-f-XmaJI | atatacctagggactcctgttgatagatccagtaat |
| AX016 | T7Te-r-Alw44I-Eco31I | atataggtctcgtgcaccgcagaaaggcccacccgaa |
| AX017 | WT-f-XbaI | atatctagaagtggccttattaaatgacttctctaagccaaaatcccttaacgtgagt |
| AX018 | WT-r-XhoI | atatctcgagttagagaagtcatttaataaggccactctcttcctttttcaattcagaa |
| AX019 | galK-LA-f-XbaI | cacttctagagcctgcaaaaagaatattttgccgaacagaaatca |
| AX020 | galK-LA-r-Eco31I | attatggtctcattcttacactccggattcgcg |
| AX021 | RFP-f-Eco31I | attatggtctcaagaaatggcttcctccgaagacg |
| AX022 | RFP-r-BamHI | tacagggatcccgcagaaaggcccacccgaaggtgagccttattaagcaccggtggagtgac |
| AX023 | galK-RA-f-BamHI | ctgcgggatccctgtaactgcgggatcatggat |
| AX024 | galK-RA-r-XhoI | tctaactcgagcgccggtcatgcgtacgcca |
| AX025 | lacZ-LA-f-XbaI | gccacttctagaggcatcgttcccactgcgatgctggtt |
| AX026 | lacZ-LA-r-Eco31I | attatggtctcaagctgtttcctgtgtgaaattgtt |
| AX027 | aadA-f-Eco31I | attatggtctccagctatgcgctcacgcaactgg |
| AX028 | aadA-r-Eco31I | attatggtctcgcaaacgcagaaaggcccacccgaaggtgagccttattatttgccgactaccttggtg |
| AX029 | lacZ-RA-f-Eco31I | attatggtctcatttgccgtctgaatttgacctg |
| AX030 | lacZ-RA-r-XhoI | tctaactcgagggaaaccgacatcgcaggcttct |
| AX031 | pyrF-LA-f-XbaI | attattctagatgctgatatcatcgaagcgc |
| AX032 | pyrF-LA-r-Eco31I | attatggtctctgaccagaccttcttgatgatgggca |
| AX033 | sfGFP-f-Eco31I | attatggtctctggtcatgcgtaaaggcgaagagct |
| AX034 | sfGFP-r-Eco31I | attatggtctcaacaccgcagaaaggcccacccgaaggtgagccttattatttgtacagttcatccataccatg |
| AX035 | pyrF-RA-f-Eco31I | attatggtctcagtgttctgctcaggaagctgtg |
| AX036 | pyrF-RA-r-XhoI | ctaactcgagttatcgccctggatttcaataact |
| AX037 | Template-f-EcoRI-SalI | atatagtcgacaatatgaattcaaaaggccaggaaccgta |
| AX038 | Template-r-MfeI | atatacaattgctgaccccggatgaatgt |
| AX039 | PJ-f-Eco31I-TCGA | atataggtctcatcgattgacagctagctcagtcctag |
| AX040 | T7Te-r-Eco31I-TCGA | atataggtctcatcgacgcagaaaggcccacccgaa |
| AX041 | Twt-r-XmaJI-Eco31I | atataggtctccctaggaaaaaggaagagagtggcct |
| AX055 | 50-pyrF-f-XbaI | tctaatctagatttacctgtttcgcgccact |
| AX056 | 50-pyrF-r-XhoI | tctaactcgagactcctgaccgaatacctgtttaa |
| AX057 | 100-pyrF-f-XbaI | tctaatctagagagaaatcgcaactgttaattttttatttcca |
| AX058 | 100-pyrF-r-XhoI | tctaactcgaggtcaccagcttcactcccct |
| AX167 | pyrF-LA-r-Eco31I-GACC | attatggtctctgaccagaccttcttgatgatgggca |
| AX168 | pyrF-RA-f-HindIII | atataaagcttgtgttctgctcaggaagctgt |

**Table S2. Primers used for plasmid construction.**

| Name | Sequence |
| --- | --- |
| SpCas9 | gtgcacgaattcaaaagatctaaagaggagaaaggatct**atggataagaaatactcaataggcttagatatcggcacaaatagcgtcggatgggcggtgatcactgatgaatataaggttccgtctaaaaagttcaaggttctgggaaatacagaccgccacagtatcaaaaaaaatcttataggggctcttttatttgacagtggagagacagcggaagcgactcgtctcaaacggacagctcgtagaaggtatacacgtcggaagaatcgtatttgttatctacaggagattttttcaaatgagatggcgaaagtagatgatagtttctttcatcgacttgaagagtcttttttggtggaagaagacaagaagcatgaacgtcatcctatttttggaaatatagtagatgaagttgcttatcatgagaaatatccaactatctatcatctgcgaaaaaaattggtagattctactgataaagcggatttgcgcttaatctatttggccttagcgcatatgattaagtttcgtggtcattttttgattgagggagatttaaatcctgataatagtgatgtggacaaactatttatccagttggtacaaacctacaatcaattatttgaagaaaaccctattaacgcaagtggagtagatgctaaagcgattctttctgcacgattgagtaaatcaagacgattagaaaatctcattgctcagctccccggtgagaagaaaaatggcttatttgggaatctcattgctttgtcattgggtttgacccctaattttaaatcaaattttgatttggcagaagatgctaaattacagctttcaaaagatacttacgatgatgatttagataatttattggcgcaaattggagatcaatatgctgatttgtttttggcagctaagaatttatcagatgctattttactttcagatatcctaagagtaaatactgaaataactaaggctcccctatcagcttcaatgattaaacgctacgatgaacatcatcaagacttgactcttttaaaagctttagttcgacaacaacttccagaaaagtataaagaaatcttttttgatcaatcaaaaaacggatatgcaggttatattgatgggggagctagccaagaagaattttataaatttatcaaaccaattttagaaaaaatggatggtactgaggaattattggtgaaactaaatcgtgaagatttgctgcgcaagcaacggacctttgacaacggctctattccccatcaaattcacttgggtgagctgcatgctattttgagaagacaagaagacttttatccatttttaaaagacaatcgtgagaagattgaaaaaatcttgacttttcgaattccttattatgttggtccattggcgcgtggcaatagtcgttttgcatggatgactcggaagtctgaagaaacaattaccccatggaattttgaagaagttgtcgataaaggtgcttcagctcaatcatttattgaacgcatgacaaactttgataaaaatcttccaaatgaaaaagtactaccaaaacatagtttgctttatgagtattttacggtttataacgaattgacaaaggtcaaatatgttactgaaggaatgcgaaaaccagcatttctttcaggtgaacagaagaaagccattgttgatttactcttcaaaacaaatcgaaaagtaaccgttaagcaattaaaagaagattatttcaaaaaaatagaatgttttgatagtgttgaaatttcaggagttgaagatagatttaatgcttcattaggtacctaccatgatttgctaaaaattattaaagataaagattttttggataatgaagaaaatgaagatatcttagaggatattgttttaacattgaccttatttgaagatagggagatgattgaggaaagacttaaaacatatgctcacctctttgatgataaggtgatgaaacagcttaaacgtcgccgttatactggttggggacgtttgtctcgaaaattgattaatggtattagggataagcaatctggcaaaacaatattagattttttgaaatcagatggttttgccaatcgcaattttatgcagctgatccatgatgatagtttgacatttaaagaagacattcaaaaagcacaagtgtctggacaaggcgatagtttacatgaacatattgcaaatttagctggtagccctgctattaaaaaaggtattttacagactgtaaaagttgttgatgaattggtcaaagtaatggggcggcataagccagaaaatatcgttattgaaatggcacgtgaaaatcagacaactcaaaagggccagaaaaattcgcgagagcgtatgaaacgaatcgaagaaggtatcaaagaattaggaagtcagattcttaaagagcatcctgttgaaaatactcaattgcaaaatgaaaagctctatctctattatctccaaaatggaagagacatgtatgtggaccaagaattagatattaatcgtttaagtgattatgatgtcgatcacattgttccacaaagtttccttaaagacgattcaatagacaataaggtcttaacgcgttctgataaaaatcgtggtaaatcggataacgttccaagtgaagaagtagtcaaaaagatgaaaaactattggagacaacttctaaacgccaagttaatcactcaacgtaagtttgataatttaacgaaagctgaacgtggaggtttgagtgaacttgataaagctggttttatcaaacgccaattggttgaaactcgccaaatcactaagcatgtggcacaaattttggatagtcgcatgaatactaaatacgatgaaaatgataaacttattcgagaggttaaagtgattaccttaaaatctaaattagtttctgacttccgaaaagatttccaattctataaagtacgtgagattaacaattaccatcatgcccatgatgcgtatctaaatgccgtcgttggaactgctttgattaagaaatatccaaaacttgaatcggagtttgtctatggtgattataaagtttatgatgttcgtaaaatgattgctaagtctgagcaagaaataggcaaagcaaccgcaaaatatttcttttactctaatatcatgaacttcttcaaaacagaaattacacttgcaaatggagagattcgcaaacgccctctaatcgaaactaatggggaaactggagaaattgtctgggataaagggcgagattttgccacagtgcgcaaagtattgtccatgccccaagtcaatattgtcaagaaaacagaagtacagacaggcggattctccaaggagtcaattttaccaaaaagaaattcggacaagcttattgctcgtaaaaaagactgggatccaaaaaaatatggtggttttgatagtccaacggtagcttattcagtcctagtggttgctaaggtggaaaaagggaaatcgaagaagttaaaatccgttaaagagttactagggatcacaattatggaaagaagttcctttgaaaaaaatccgattgactttttagaagctaaaggatataaggaagttaaaaaagacttaatcattaaactacctaaatatagtctttttgagttagaaaacggtcgtaaacggatgctggctagtgccggagaattacaaaaaggaaatgagctggctctgccaagcaaatatgtgaattttttatatttagctagtcattatgaaaagttgaagggtagtccagaagataacgaacaaaaacaattgtttgtggagcagcataagcattatttagatgagattattgagcaaatcagtgaattttctaagcgtgttattttagcagatgccaatttagataaagttcttagtgcatataacaaacatagagacaaaccaatacgtgaacaagcagaaaatattattcatttatttacgttgacgaatcttggagctcccgctgcttttaaatattttgatacaacaattgatcgtaaacgatatacgtctacaaaagaagttttagatgccactcttatccatcaatccatcactggtctttatgaaacacgcattgatttgagtcagctaggaggtgactaa**ctcgagtaaggatctccaggcatcaaataaaacgaaaggctcagtcgaaagactgggcctttcgttttatctgttgtttgtcggtgaacgctctctactagagtcacactggctcaccttcgggtgggcctttctgcgtttatacggccgcctgcaggtcccgggggcatgcgtcgacatatagcggccg |
| FnCas12a | gtgcacgaattcaaaagatctaaagaggagaaaggatct**atgtcaatttatcaagaatttgttaataaatatagtttaagtaaaactctaagatttgagttaatcccacagggtaaaacacttgaaaacataaaagcaagaggtttgattttagatgatgagaaaagagctaaagactacaaaaaggctaaacaaataattgataaatatcatcagttttttatagaggagatattaagttcggtttgtattagcgaagatttattacaaaactattctgatgtttattttaaacttaaaaagagtgatgatgataatctacaaaaagattttaaaagtgcaaaagatacgataaagaaacaaatatctgaatatataaaggactcagagaaatttaagaatttgtttaatcaaaaccttatcgatgctaaaaaagggcaagagtcagatttaattctatggctaaagcaatctaaggataatggtatagaactatttaaagccaatagtgatatcacagatatagatgaggcgttagaaataatcaaatcttttaaaggttggacaacttattttaagggttttcatgaaaatagaaaaaatgtttatagtagcaatgatattcctacatctattatttataggatagtagatgataatttgcctaaatttctagaaaataaagctaagtatgagagtttaaaagacaaagctccagaagctataaactatgaacaaattaaaaaagatttggcagaagagctaacctttgatattgactacaaaacatctgaagttaatcaaagagttttttcacttgatgaagtttttgagatagcaaactttaataattatctaaatcaaagtggtattactaaatttaatactattattggtggtaaatttgtaaatggtgaaaatacaaagagaaaaggtataaatgaatatataaatctatactcacagcaaataaatgataaaacactcaaaaaatataaaatgagtgttttatttaagcaaattttaagtgatacagaatctaaatcttttgtaattgataagttagaagatgatagtgatgtagttacaacgatgcaaagtttttatgagcaaatagcagcttttaaaacagtagaagaaaaatctattaaagaaacactatctttattatttgatgatttaaaagctcaaaaacttgatttgagtaaaatttattttaaaaatgataaatctcttactgatctatcacaacaagtttttgatgattatagtgttattggtacagcggtactagaatatataactcaacaaatagcacctaaaaatcttgataaccctagtaagaaagagcaagaattaatagccaaaaaaactgaaaaagcaaaatacttatctctagaaactataaagcttgccttagaagaatttaataagcatagagatatagataaacagtgtaggtttgaagaaatacttgcaaactttgcggctattccgatgatatttgatgaaatagctcaaaacaaagacaatttggcacagatatctatcaaatatcaaaatcaaggtaaaaaagacctacttcaagctagtgcggaagatgatgttaaagctatcaaggatcttttagatcaaactaataatctcttacataaactaaaaatatttcatattagtcagtcagaagataaggcaaatattttagacaaggatgagcatttttatctagtatttgaggagtgctactttgagctagcgaatatagtgcctctttataacaaaattagaaactatataactcaaaagccatatagtgatgagaaatttaagctcaattttgagaactcgactttggctaatggttgggataaaaataaagagcctgacaatacggcaattttatttatcaaagatgataaatattatctgggtgtgatgaataagaaaaataacaaaatatttgatgataaagctatcaaagaaaataaaggcgagggttataaaaaaattgtttataaacttttacctggcgcaaataaaatgttacctaaggttttcttttctgctaaatctataaaattttataatcctagtgaagatatacttagaataagaaatcattccacacatacaaaaaatggtagtcctcaaaaaggatatgaaaaatttgagtttaatattgaagattgccgaaaatttatagatttttataaacagtctataagtaagcatccggagtggaaagattttggatttagattttctgatactcaaagatataattctatagatgaattttatagagaagttgaaaatcaaggctacaaactaacttttgaaaatatatcagagagctatattgatagcgtagttaatcagggtaaattgtacctattccaaatctataataaagatttttcagcttatagcaaagggcgaccaaatctacatactttatattggaaagcgctgtttgatgagagaaatcttcaagatgtggtttataagctaaatggtgaggcagagcttttttatcgtaaacaatcaatacctaaaaaaatcactcacccagctaaagaggcaatagctaataaaaacaaagataatcctaaaaaagagagtgtttttgaatatgatttaatcaaagataaacgctttactgaagataagtttttctttcactgtcctattacaatcaattttaaatctagtggagctaataagtttaatgatgaaatcaatttattgctaaaagaaaaagcaaatgatgttcatatattaagtatagatagaggtgaaagacatttagcttactatactttggtagatggtaaaggcaatatcatcaaacaagatactttcaacatcattggtaatgatagaatgaaaacaaactaccatgataagcttgctgcaatagagaaagatagggattcagctaggaaagactggaaaaagataaataacatcaaagagatgaaagagggctatctatctcaggtagttcatgaaatagctaagctagttatagagtataatgctattgtggtttttgaggatttaaattttggatttaaaagagggcgtttcaaggtagagaagcaggtctatcaaaagttagaaaaaatgctaattgagaaactaaactatctagttttcaaagataatgagtttgataaaactgggggagtgcttagagcttatcagctaacagcaccttttgagacttttaaaaagatgggtaaacaaacaggtattatctactatgtaccagctggttttacttcaaaaatttgtcctgtaactggttttgtaaatcagttatatcctaagtatgaaagtgtcagcaaatctcaagagttctttagtaagtttgacaagatttgttataaccttgataagggctattttgagtttagttttgattataaaaactttggtgacaaggctgccaaaggcaagtggactatagctagctttgggagtagattgattaactttagaaattcagataaaaatcataattgggatactcgagaagtttatccaactaaagagttggagaaattgctaaaagattattctatcgaatatgggcatggcgaatgtatcaaagcagctatttgcggtgagagcgacaaaaagttttttgctaagctaactagtgtcctaaatactatcttacaaatgcgtaactcaaaaacaggtactgagttagattatctaatttcaccagtagcagatgtaaatggcaatttctttgattcgcgacaggcgccaaaaaatatgcctcaagatgctgatgccaatggtgcttatcatattgggctaaaaggtctgatgctactaggtaggatcaaaaataatcaagagggcaaaaaactcaatttggttatcaaaaatgaagagtattttgagttcgtgcagaataggaataactaa**ccaggcatcaaataaaacgaaaggctcagtcgaaagactgggcctttcgttttatctgttgtttgtcggtgaacgctctctactagagtcacactggctcaccttcgggtgggcctttctgcgggtctcaatatacccgggggcatgc |
| codon optimized FnCas12a (type 1) | gtgcacgaattcaaaagatctaaagaggagaaaggatct**atgtccatctaccaggagttcgtaaacaaatactccctgtccaaaacgctgcgtttcgaactgatcccgcagggtaagaccctcgaaaacattaaagcccgtggtctgatcctggacgatgaaaaacgtgcaaaggattacaaaaaagctaaacagatcatcgataagtatcaccaattcttcattgaagagatcctgtcttctgtctgtattagcgaagacctgctgcaaaactattccgatgtgtacttcaaactgaaaaaatccgacgatgacaacctgcagaaagacttcaagagcgcaaaagacactatcaaaaaacaaatttccgaatatatcaaagacagcgaaaaattcaaaaacctgtttaaccaaaacctgatcgatgcgaaaaagggccaggaaagtgatctgatcctgtggctgaaacagtccaaagataacggcatcgaactgtttaaagccaactctgatatcaccgatatcgatgaggcactggaaatcatcaaatctttcaaaggctggactacctacttcaaaggtttccacgaaaaccgtaagaacgtatactcctcgaatgacatcccgacttctatcatttatcgtatcgtggatgataacctgccaaaattcctggaaaacaaagctaaatacgagtccctgaaagataaagcgccggaagctatcaactatgagcaaattaaaaaagacctggcggaagagctgacttttgacatcgattacaaaacttctgaggttaaccagcgtgtattctccctggatgaagttttcgaaatcgcaaatttcaacaactatctgaaccagagtggtatcaccaaattcaacaccatcatcggtggcaaattcgttaacggtgaaaacaccaaacgtaaaggtatcaacgaatatatcaatctgtattcccagcagattaacgacaaaacactgaaaaaatataaaatgtcggtgctgttcaaacagatcctgtccgacacggaaagcaaatcattcgttattgataaactcgaagacgactctgatgtagtgaccactatgcagagcttctacgaacagattgcggcttttaaaaccgtggaggaaaaatctattaaagaaaccctgagcctgctgtttgacgacctgaaggcgcagaagctggacctttctaaaatctactttaaaaacgataaatctctgaccgacctgagccagcaggtatttgatgactactctgttatcggtaccgcagtactggaatacatcacccagcagattgccccgaaaaacctggataatccatctaaaaaagaacaggaactgattgctaaaaaaaccgaaaaagcgaaatacctgtctttggaaacgattaaactggcgctcgaagaatttaacaaacaccgtgacatcgataaacagtgccgcttcgaggagatcctggccaacttcgcagcgatcccgatgatcttcgacgaaatcgcgcagaacaaggataacctggcgcagatctcaattaaataccagaaccagggcaagaaagatctgctgcaggcgagtgcggaagacgatgtgaaagccatcaaagacctgttggaccaaactaataacctgctgcataaactgaagatttttcatatcagtcagtccgaagataaagcgaacattctggacaaagacgaacacttctatctggtatttgaggaatgctatttcgagctggcgaacattgttccgctgtacaataagattcgtaactacatcacccagaagccgtattccgatgaaaaattcaaactgaacttcgaaaattcaactttggcgaacggctgggataagaacaaagagccggacaacacggccattctcttcatcaaagatgacaaatactacctgggtgtgatgaataagaaaaataacaaaatcttcgacgataaagcgatcaaagaaaacaaaggtgaaggttacaaaaaaatcgtttataaactgctgccgggcgcgaacaaaatgctgccgaaagtttttttcagtgcgaaatctatcaagttctataacccgtctgaagatatcttgcgtatccgtaaccactccactcataccaaaaacggctccccgcaaaaaggctatgagaaatttgaattcaacattgaagattgccgtaaattcattgacttctacaaacagtcaatctccaaacacccggaatggaaagacttcggcttccgcttctctgatactcagcgttacaactctatcgatgaattttaccgtgaagtggaaaaccagggctacaagcttaccttcgaaaacatttctgagagctatatcgattcggttgttaaccaggggaaactgtatctttttcaaatctataacaaagatttctctgcctactccaaaggccgcccaaacctgcacaccctgtattggaaagctctgtttgacgaacgtaacctgcaggacgtagtgtacaagctgaacggtgaggcagaactgttttatcgtaaacagtccatcccaaaaaagattactcatccggccaaagaagcgatcgcgaacaaaaacaaagacaacccgaagaaggaatcagttttcgaatatgacctgattaaagataaacgtttcactgaggacaaatttttcttccattgtccgatcactatcaacttcaaatctagcggcgcaaacaaattcaacgacgagattaatctgctcctgaaagagaaagcgaacgatgtacacattttgtctatcgaccgtggggagcgtcacctggcttactacaccctggtggacggcaagggtaatatcatcaagcaggatactttcaacatcatcgggaacgatcgtatgaaaaccaactatcatgataaactggctgcaatcgagaaagaccgtgattctgcgcgtaaagattggaagaaaatcaacaacatcaaagaaatgaaagagggctacttgagccaagttgttcacgaaattgcgaaactggttatcgaatacaatgcgatcgttgtgttcgaggatctgaacttcggttttaagcgtggccgcttcaaagtggaaaaacaggtttaccaaaaattagaaaaaatgctgatcgaaaaactgaactacctggtattcaaagataacgaatttgacaaaactggtggcgttctgcgtgcataccagctcaccgcgccgttcgaaactttcaaaaaaatgggcaaacagactggtattatctattatgtaccggccggtttcacctcaaaaatctgcccggttaccggcttcgttaaccagctgtacccgaaatacgagtccgtatccaaatctcaggagttcttctctaagttcgacaaaatttgctacaacctggacaaaggttatttcgaattctccttcgactacaaaaatttcggtgacaaggcggcaaaaggtaaatggacgattgcttctttcggctctcgtctgatcaacttccgtaattctgataaaaatcataactgggacacccgcgaagtatatccaaccaaggagctggagaaactgctgaaagattattccatcgaatatggtcacggtgaatgtatcaaagcggcaatttgcggtgaatctgacaagaaattcttcgcgaaactgacctctgtcctgaacaccatcctgcagatgcgtaacagcaaaaccggtaccgaactggactacctgatttctccagttgcggacgttaatggcaacttttttgactcccgtcaggctccgaagaacatgccgcaggacgctgatgcgaacggtgcctatcacatcggtctgaaaggtctgatgttgctgggccgtatcaaaaacaatcaggaaggcaaaaaactgaacctggtgatcaaaaacgaggaatatttcgaattcgttcagaaccgcaacaactaa**ccaggcatcaaataaaacgaaaggctcagtcgaaagactgggcctttcgttttatctgttgtttgtcggtgaacgctctctactagagtcacactggctcaccttcgggtgggcctttctgcgggtctcaatatacccgggggcatgc |
| codon optimized FnCas12a (type 2) | gtgcacgaattcaaaagatctaaagaggagaaaggatct**atgtctatctaccaggaattcgttaacaaatactctctgtctaaaaccctgcgtttcgaactgatcccgcagggtaaaaccctggaaaacatcaaagctcgtggtctgatcctggacgacgaaaaacgtgctaaagactacaaaaaagctaaacagatcatcgacaaataccaccagttcttcatcgaagaaatcctctcgtctgtctgcatcagcgaagacctgctgcagaactactcggacgtgtacttcaaactaaaaaaatctgacgacgacaacctgcagaaagacttcaaatctgctaaagacaccatcaaaaaacagatctctgaatacatcaaagactctgaaaaattcaaaaacctgttcaaccagaacctgatcgacgctaaaaaaggtcaggaatctgacctgatcctgtggctgaaacagtctaaagacaacggtatcgaactgttcaaagctaactctgacatcaccgacatcgacgaagctctggaaatcatcaaatctttcaaaggttggaccacctacttcaaaggtttccacgaaaaccgtaaaaacgtttactcttctaacgacatcccgacctctatcatctaccgtatcgttgacgacaacctgccgaaattcctggaaaacaaagctaaatacgaatctctgaaagacaaagctccggaagctatcaactacgaacagatcaaaaaagacctggctgaagaactgaccttcgacatcgactacaaaacctctgaagttaaccagcgtgttttctctctggacgaagttttcgaaatcgctaacttcaacaactacctgaaccagtctggtatcaccaaattcaacaccatcatcggtggtaaattcgttaacggtgaaaacaccaaacgtaaaggtatcaacgaatacatcaacctgtactctcagcagatcaacgacaaaaccctgaaaaaatacaaaatgtctgttctgttcaaacagatcctgtctgacaccgaatctaaatctttcgttatcgacaaactggaagacgactctgacgttgttaccaccatgcagtctttctacgaacagatcgctgctttcaaaaccgttgaagaaaaatctatcaaagaaaccctgtctctgctgttcgacgacctgaaagctcagaaactggacctgtctaaaatctacttcaaaaacgacaaatctctgaccgacctctctcagcaggtattcgacgactacagcgttatcggtaccgctgttctggaatacatcacccagcagatcgctccgaaaaacctggacaacccgtctaaaaaagaacaggaactgatcgctaaaaaaaccgaaaaagctaaatacctgtctctggaaaccatcaaactggctctggaagaattcaacaaacaccgtgacatcgacaaacagtgccgtttcgaagaaatcctggctaacttcgctgctatcccgatgatcttcgacgaaatcgctcagaacaaagacaacctggctcagatctctatcaaataccagaaccagggtaaaaaagacctgctgcaggcttctgctgaagacgacgttaaagctatcaaagacctgctggaccagaccaacaacctgctgcacaaactgaaaatcttccacatctctcagtctgaagacaaagctaacatcctggacaaagacgaacacttctacctggttttcgaagaatgctacttcgaactggctaacatcgttccgctgtacaacaaaatccgtaactacatcacccagaaaccgtactctgacgaaaaattcaaactgaacttcgaaaactctaccctggctaacggttgggacaaaaacaaagaaccggacaacaccgctatcctgttcatcaaagacgacaaatactacctgggtgttatgaacaaaaaaaacaacaaaatcttcgacgacaaagctatcaaagaaaacaaaggtgaaggttacaaaaaaatcgtttacaaactgctgccgggtgctaacaaaatgctgccgaaagttttcttctctgctaaatctatcaaattctacaacccgtctgaagacatcctgcgtatccgtaaccactctacccacaccaaaaacggttctccgcagaaaggttacgaaaaattcgaattcaacatcgaagactgccgtaaattcatcgacttctacaaacagtctatctctaaacacccggaatggaaagacttcggtttccgtttctctgacacccagcgttacaactctatcgacgaattctaccgtgaagttgaaaaccagggttacaaactgaccttcgaaaacatctctgaatcttacatcgactctgttgttaaccagggtaaactgtacctgttccagatctacaacaaagacttctctgcttactctaaaggtcgtccgaacctgcacaccctgtactggaaagctctgttcgacgaacgtaacctgcaggacgttgtttacaaactgaacggtgaagctgaactgttctaccgtaaacagtctatcccgaaaaaaatcacccacccggctaaagaagctatcgctaacaaaaacaaagacaacccgaaaaaagaatctgttttcgaatacgacctgatcaaagacaaacgtttcaccgaagacaaattcttcttccactgcccgatcaccatcaacttcaaatcttctggtgctaacaaattcaacgacgaaatcaacctgctgctgaaagaaaaagctaacgacgttcacatcctgtctatcgaccgtggtgaacgtcacctggcttactacaccctggttgacggtaaaggtaacatcatcaaacaggacaccttcaacatcatcggtaacgaccgtatgaaaaccaactaccacgacaaactggctgctatcgaaaaagaccgtgactctgctcgtaaagactggaaaaaaatcaacaacatcaaagaaatgaaagaaggttacctgtctcaggttgttcacgaaatcgctaaactggttatagaatacaatgcgatagttgtatttgaagacctgaacttcggcttcaaacgtggtcgtttcaaagttgaaaaacaggtttaccagaaactggaaaaaatgctgatcgaaaaactgaactacctggttttcaaagacaacgaattcgacaaaaccggtggtgttctgcgtgcttaccagctgaccgctccgttcgaaaccttcaaaaaaatgggtaaacagaccggtatcatctactacgttccggctggtttcacctctaaaatctgccctgttactggcttcgttaaccagctctacccgaaatacgaatctgtttctaaatctcaggaattcttctctaaattcgacaaaatctgctacaacctggacaaaggttacttcgaattctctttcgactacaaaaacttcggtgacaaagctgctaaaggtaaatggaccatcgcttctttcggttctcgtctgatcaacttccgtaactctgacaaaaaccacaactgggacacccgtgaagtttacccgaccaaagaactggaaaaactgctgaaagactactctatcgaatacggtcacggtgaatgcatcaaagctgctatctgcggtgaatctgacaaaaaattcttcgctaaactgacctctgttctgaacaccatcctgcagatgcgtaactctaaaaccggtaccgaactggactacctgatctctccggttgctgacgttaacggtaacttcttcgactctcgtcaggctccgaaaaacatgccgcaggacgctgacgctaacggtgcttaccacatcggtctgaaaggtctgatgctgctgggtcgtatcaaaaacaaccaggaaggtaaaaaactgaacctggttatcaaaaacgaagaatacttcgaattcgttcagaaccgtaacaactaa**ccaggcatcaaataaaacgaaaggctcagtcgaaagactgggcctttcgttttatctgttgtttgtcggtgaacgctctctactagagtcacactggctcaccttcgggtgggcctttctgcgggtctcaatatacccgggggcatgc |
| crRNA-araD | gtcgacTTGACAGCTAGCTCAGTCCTAGGTATAATACTAGTtcgagattttcaggagctaaggaagctaaa**GTCTAAGAACTTTAAATAATTTCTACTGTTGTAGATGAAGCCAACCTGGCGCTGCCAAAACACAACCGTCTAAGAACTTTAAATAATTTCTACTGTTGTAGAT**gagaagtcatttaataaggccactggctcaccttcgggtgggcctttctgcgcaattg |
| crRNA-araD2 | gtcgacTTGACAGCTAGCTCAGTCCTAGGTATAATACTAGTtcgagattttcaggagctaaggaagctaaa**GTCTAAGAACTTTAAATAATTTCTACTGTTGTAGATAACCACTTCACCGGTTTCGATGCTAACCACGGTCTAAGAACTTTAAATAATTTCTACTGTTGTAGAT**gagaagtcatttaataaggccactggctcaccttcgggtgggcctttctgcgcaattg |
| crRNA-galK | gtcgacTTGACAGCTAGCTCAGTCCTAGGTATAATACTAGTtcgagattttcaggagctaaggaagctaaa**GTCTAAGAACTTTAAATAATTTCTACTGTTGTAGATCCAACGCATTTGGCTACCCTGCCACTCACACGTCTAAGAACTTTAAATAATTTCTACTGTTGTAGAT**gagaagtcatttaataaggccactggctcaccttcgggtgggcctttctgcgcaattg |
| crRNA-galK2 | gtcgacTTGACAGCTAGCTCAGTCCTAGGTATAATACTAGTtcgagattttcaggagctaaggaagctaaa**GTCTAAGAACTTTAAATAATTTCTACTGTTGTAGATACGGTCATCGCGTGGTGCACAACTGATCACGGTCTAAGAACTTTAAATAATTTCTACTGTTGTAGAT**gagaagtcatttaataaggccactggctcaccttcgggtgggcctttctgcgcaattg |
| crRNA-lacZ | gtcgacTTGACAGCTAGCTCAGTCCTAGGTATAATACTAGTtcgagattttcaggagctaaggaagctaaa**GTCTAAGAACTTTAAATAATTTCTACTGTTGTAGATCGGATTCACTGGCCGTCGTTTTACAACGTCGGTCTAAGAACTTTAAATAATTTCTACTGTTGTAGAT**gagaagtcatttaataaggccactggctcaccttcgggtgggcctttctgcgcaattg |
| crRNA-mutS | gtcgacTTGACAGCTAGCTCAGTCCTAGGTATAATACTAGTtcgagattttcaggagctaaggaagctaaa**GTCTAAGAACTTTAAATAATTTCTACTGTTGTAGATCGCTCAACCGGACCTTTGCTGGTCGCCGGATGTCTAAGAACTTTAAATAATTTCTACTGTTGTAGAT**gagaagtcatttaataaggccactggctcaccttcgggtgggcctttctgcgcaattg |
| crRNA-pyrF | gtcgacTTGACAGCTAGCTCAGTCCTAGGTATAATACTAGTtcgagattttcaggagctaaggaagctaaa**GTCTAAGAACTTTAAATAATTTCTACTGTTGTAGATTTCCCGCGCTGTTACGAATTCTCCTGTGGTTGTCTAAGAACTTTAAATAATTTCTACTGTTGTAGAT**gagaagtcatttaataaggccactggctcaccttcgggtgggcctttctgcgcaattg |
| sgRNA-galK | gtcgacTTGACAGCTAGCTCAGTCCTAGGTATAATACTAGT**TCTCTGTTTGCCAACGCATTGTTTTAGAGCTAGAAATAGCAAGTTAAAATAAGGCTAGTCCGTTATCAACTTGAAAAAGTGGCACCGAGTCGGTGCTTTTTTTG**ggctcaccttcgggtgggcctttctgcgcaattg |
| sgRNA-lacZ | gtcgacTTGACAGCTAGCTCAGTCCTAGGTATAATACTAGT**TCGTTTTACAACGTCGTGACGTTTTAGAGCTAGAAATAGCAAGTTAAAATAAGGCTAGTCCGTTATCAACTTGAAAAAGTGGCACCGAGTCGGTGCTTTTTTTG**ggctcaccttcgggtgggcctttctgcgcaattg |
| sgRNA-pyrF | gtcgacTTGACAGCTAGCTCAGTCCTAGGTATAATACTAGT**TGAAGTTCGCGCACAAACTGGTTTTAGAGCTAGAAATAGCAAGTTAAAATAAGGCTAGTCCGTTATCAACTTGAAAAAGTGGCACCGAGTCGGTGCTTTTTTTG**ggctcaccttcgggtgggcctttctgcgcaattg |
| crRNA-lacZ-pyrF | gtcgacTTGACAGCTAGCTCAGTCCTAGGTATAATACTAGTtcgagattttcaggagctaaggaagctaaa**GTCTAAGAACTTTAAATAATTTCTACTGTTGTAGATCGGATTCACTGGCCGTCGTTTTACAACGTCGGTCTAAGAACTTTAAATAATTTCTACTGTTGTAGATTTCCCGCGCTGTTACGAATTCTCCTGTGGTTGTCTAAGAACTTTAAATAATTTCTACTGTTGTAGAT**gagaagtcatttaataaggccactggctcaccttcgggtgggcctttctgcgcaattg |
| crRNA-pyrF-lacZ | gtcgacTTGACAGCTAGCTCAGTCCTAGGTATAATACTAGTtcgagattttcaggagctaaggaagctaaa**GTCTAAGAACTTTAAATAATTTCTACTGTTGTAGATTTCCCGCGCTGTTACGAATTCTCCTGTGGTTGTCTAAGAACTTTAAATAATTTCTACTGTTGTAGATCGGATTCACTGGCCGTCGTTTTACAACGTCGGTCTAAGAACTTTAAATAATTTCTACTGTTGTAGAT**gagaagtcatttaataaggccactggctcaccttcgggtgggcctttctgcgcaattg |
| crRNA-torS | gtcgacTTGACAGCTAGCTCAGTCCTAGGTATAATACTAGTtcgagattttcaggagctaaggaagctaaa**GTCTAAGAACTTTAAATAATTTCTACTGTTGTAGATAGGTGAAAAAGGTTGAGTCGCAAAGCGGAATGTCTAAGAACTTTAAATAATTTCTACTGTTGTAGAT**gagaagtcatttaataaggccactggctcaccttcgggtgggcctttctgcgcaattg |
| crRNA-prpC | gtcgacTTGACAGCTAGCTCAGTCCTAGGTATAATACTAGTtcgagattttcaggagctaaggaagctaaa**GTCTAAGAACTTTAAATAATTTCTACTGTTGTAGATCTACATATGCTGCGCGGTGAGCCTGCTTCTGGTCTAAGAACTTTAAATAATTTCTACTGTTGTAGAT**gagaagtcatttaataaggccactggctcaccttcgggtgggcctttctgcgcaattg |

**Table S3. Synthetic sequences.** Sequences of Cas protein genes were shown in bold lowercase letters. Sequences of unprocessed crRNA and sgRNA were shown in bold uppercase letters. Sequences of the J23119(SpeI) promoter were shown in normal uppercase letters.

| no. | name | SEQUENCE | | | BINDING SITE |
| --- | --- | --- | --- | --- | --- |
| AX042 | galK-524-f | | ctaacgaagctgagcgcgaaga | Upstream of *galK*’s LA | |
| AX043 | RFP-NR | | ctttgataacgtcttcggaggaagccat | *rfp* | |
| AX044 | galK-225+r | | gagctgattttcataatcggctgcca | *galK* | |
| AX045 | galK-68-f | | cagaacaggcagcagagcgtt | *galK*’s LA | |
| AX046 | galK-41+r | | gcgttggcaaacagagattgtgttt | *galK* | |
| AX047 | lacZ-533-f | | ggttttcaacaaaccatgcaaatgctgaatga | Upstream of *lacZ*’s LA | |
| AX048 | aadA-NR | | gaccagttgcgtgagcgcat | *aadA* | |
| AX049 | lacZ-80+r | | cgattaagttgggtaacgccagggtt | *lacZ* | |
| AX050 | lacZ-477+f | | gtcggttacggccaggacagt | *lacZ* | |
| AX051 | lacZ-777+r | | aactgttacccgtaggtagtcacgca | *lacZ*’s RA | |
| AX052 | pyrF-520-f | | gccgatgctgaattgatgct | Upstream of *pyrF*’s LA | |
| AX053 | GFP-NR | | tgaacagctcttcgcctttacgcat | *gfp* | |
| AX054 | pyrF-29+r | | cgggaagaagatgaagcagttaacgtcat | *pyrF* | |
| AX059 | araD-529-f | | cgaaactgccggtggcgaat | Upstream of *araD*’s LA | |
| AX060 | RPSL-NR | | ccagctggttaactgttgccat | *rpsl* | |
| AX061 | araD-34+r | | cttctaatacctggcgtttgagatcttctaacat | *araD* | |
| AX062 | mutS-527-f | | cggcccaacgcataatctgtttcatct | Upstream of *mutS*’s LA | |
| AX063 | TCR-NR | | acgatgagcgcattgttagatttcat | *tcr* | |
| AX064 | mutS-81+r | | gatctcgggatgctgggctttca | *mutS* | |
| AX066 | pyrF-1053+r | | cgagttttaccttcatccctttcgcttcaa | Downstream of *pyrF*’s RA | |
| AX067 | torS-YZ-F1 | | atcaaatcgtaaatcccggcct | Upstream of *torS*’s LA | |
| AX068 | torS-29+r | | ctttgcgactcaacctttttcacct | *torS* | |
| AX069 | torT-YZ-Rwx | | ccgggcatcaacagcacattca | Downstream of *torS*’s RA | |
| AX070 | hem1-CF | | cctcttctcagctgtctctgacca | *hem1* | |
| AX071 | CY390 | | ttgaccgcattaaggccgca | Upstream of *prpC*’s LA | |
| AX072 | prpC-1335+r | | cgtaggcaccacaggattcactt | Downstream of *prpC*’s RA | |
| AX073 | prpC-mut-test-r | | accgcgcagcatatgtagTT | The mutant target of crRNA-prpC | |
| AX074 | prpC-test-r | | accgcgcagcatatgtagga | Target of crRNA-prpC | |

**Table S4. Primers used for genotyping.** LA, the left arm for homologous recombination. RA, the right arm for homologous recombination.

| Sites | Sequences |
| --- | --- |
| *ΔtorS*::*p103-hem1* | ATGCCTCCACACCGCTCGTCACAtcctgttgcgtTCACTGGAATCCCAgtatAGCAtTTGACCTGCGAGCAaGCTGTCACCGGATGTGCTTTCCGGTCTGATGAGTCCGTGAGGACGAAACAGCCTCTACAAATAATTTTGTTTAAgagttactagagaaagaggagaaatactagATGCAGCGTTCTATCTTCGCGCGTTTCGGTAACTCTTCTGCGGCGGTTTCTACCCTGAACCGTCTGTCTACCACCGCGGCGCCGCACGCGAAAAACGGTTACGCGACCGCGACCGGTGCGGGTGCGGCGGCGGCGACCGCGACCGCGTCTTCTACCCACGCGGCGGCGGCGGCGGCGGCGGCGGCGAACCACTCTACCCAGGAATCTGGTTTCGACTACGAAGGTCTGATCGACTCTGAACTGCAGAAAAAACGTCTGGACAAATCTTACCGTTACTTCAACAACATCAACCGTCTGGCGAAAGAATTCCCGCTGGCGCACCGTCAGCGTGAAGCGGACAAAGTTACCGTTTGGTGCTCTAACGACTACCTGGCGCTGTCTAAACACCCGGAAGTTCTGGACGCGATGCACAAAACCATCGACAAATACGGTTGCGGTGCGGGTGGTACCCGTAACATCGCGGGTCACAACATCCCGACCCTGAACCTGGAAGCGGAACTGGCGACCCTGCACAAAAAAGAAGGTGCGCTGGTTTTCTCTTCTTGCTACGTTGCGAACGACGCGGTTCTGTCTCTGCTGGGTCAGAAAATGAAAGACCTGGTTATCTTCTCTGACGAACTGAACCACGCGTCTATGATCGTTGGTATCAAACACGCGAACGTTAAAAAACACATCTTCAAACACAACGACCTGAACGAACTGGAACAGCTGCTGCAGTCTTACCCGAAATCTGTTCCGAAACTGATCGCGTTCGAATCTGTTTACTCTATGGCGGGTTCTGTTGCGGACATCGAAAAAATCTGCGACCTGGCGGACAAATACGGTGCGCTGACCTTCCTGGACGAAGTTCACGCGGTTGGTCTGTACGGTCCGCACGGTGCGGGTGTTGCGGAACACTGCGACTTCGAATCTCACCGTGCGTCTGGTATCGCGACCCCGAAAACCAACGACAAAGGTGGTGCGAAAACCGTTATGGACCGTGTTGACATGATCACCGGTACCCTGGGTAAATCTTTCGGTTCTGTTGGTGGTTACGTTGCGGCGTCTCGTAAACTGATCGACTGGTTCCGTTCTTTCGCGCCGGGTTTCATCTTCACCACCACCCTGCCGCCGTCTGTTATGGCGGGTGCGACCGCGGCGATCCGTTACCAGCGTTGCCACATCGACCTGCGTACCTCTCAGCAGAAACACACCATGTACGTTAAAAAAGCGTTCCACGAACTGGGTATCCCGGTTATCCCGAACCCGTCTCACATCGTTCCGGTTCTGATCGGTAACGCGGACCTGGCGAAACAGGCGTCTGACATCCTGATCAACAAACACCAGATCTACGTTCAGGCGATCAACTTCCCGACCGTTGCGCGTGGTACCGAACGTCTGCGTATCACCCCGACCCCGGGTCACACCAACGACCTGTCTGACATCCTGATCAACGCGGTTGACGACGTTTTCAACGAACTGCAGCTGCCGCGTGTTCGTGACTGGGAATCTCAGGGTGGTCTGCTGGGTGTTGGTGAATCTGGTTTCGTTGAAGAATCTAACCTGTGGACCTCTTCTCAGCTGTCTCTGACCAACGACGACCTGAACCCGAACGTTCGTGACCCGATCGTTAAACAGCTGGAAGTTTCTTCTGGTATCAAACAGTAA |
| *ΔtorS*::*pT7-hem1* | atgcTAATACGACTCACTATAGGgggttccccccgtaggggggtactctatgtagtagcaaccattactggaggcaacatgccacgtttacctaggtaataatcctctacaaataattttgtttaatcggTTAAGAAGGAGATATACatATGGGCAGCAGCCATCATCATCATCATCACAGCAGCGGCCTGGTGCCGCGCGGCAGCCATATGGCTAGCATGACTGGTGGACAGCAAATGGGTCGCGGATCCATGCAGCGTTCTATCTTCGCGCGTTTCGGTAACTCTTCTGCGGCGGTTTCTACCCTGAACCGTCTGTCTACCACCGCGGCGCCGCACGCGAAAAACGGTTACGCGACCGCGACCGGTGCGGGTGCGGCGGCGGCGACCGCGACCGCGTCTTCTACCCACGCGGCGGCGGCGGCGGCGGCGGCGGCGAACCACTCTACCCAGGAATCTGGTTTCGACTACGAAGGTCTGATCGACTCTGAACTGCAGAAAAAACGTCTGGACAAATCTTACCGTTACTTCAACAACATCAACCGTCTGGCGAAAGAATTCCCGCTGGCGCACCGTCAGCGTGAAGCGGACAAAGTTACCGTTTGGTGCTCTAACGACTACCTGGCGCTGTCTAAACACCCGGAAGTTCTGGACGCGATGCACAAAACCATCGACAAATACGGTTGCGGTGCGGGTGGTACCCGTAACATCGCGGGTCACAACATCCCGACCCTGAACCTGGAAGCGGAACTGGCGACCCTGCACAAAAAAGAAGGTGCGCTGGTTTTCTCTTCTTGCTACGTTGCGAACGACGCGGTTCTGTCTCTGCTGGGTCAGAAAATGAAAGACCTGGTTATCTTCTCTGACGAACTGAACCACGCGTCTATGATCGTTGGTATCAAACACGCGAACGTTAAAAAACACATCTTCAAACACAACGACCTGAACGAACTGGAACAGCTGCTGCAGTCTTACCCGAAATCTGTTCCGAAACTGATCGCGTTCGAATCTGTTTACTCTATGGCGGGTTCTGTTGCGGACATCGAAAAAATCTGCGACCTGGCGGACAAATACGGTGCGCTGACCTTCCTGGACGAAGTTCACGCGGTTGGTCTGTACGGTCCGCACGGTGCGGGTGTTGCGGAACACTGCGACTTCGAATCTCACCGTGCGTCTGGTATCGCGACCCCGAAAACCAACGACAAAGGTGGTGCGAAAACCGTTATGGACCGTGTTGACATGATCACCGGTACCCTGGGTAAATCTTTCGGTTCTGTTGGTGGTTACGTTGCGGCGTCTCGTAAACTGATCGACTGGTTCCGTTCTTTCGCGCCGGGTTTCATCTTCACCACCACCCTGCCGCCGTCTGTTATGGCGGGTGCGACCGCGGCGATCCGTTACCAGCGTTGCCACATCGACCTGCGTACCTCTCAGCAGAAACACACCATGTACGTTAAAAAAGCGTTCCACGAACTGGGTATCCCGGTTATCCCGAACCCGTCTCACATCGTTCCGGTTCTGATCGGTAACGCGGACCTGGCGAAACAGGCGTCTGACATCCTGATCAACAAACACCAGATCTACGTTCAGGCGATCAACTTCCCGACCGTTGCGCGTGGTACCGAACGTCTGCGTATCACCCCGACCCCGGGTCACACCAACGACCTGTCTGACATCCTGATCAACGCGGTTGACGACGTTTTCAACGAACTGCAGCTGCCGCGTGTTCGTGACTGGGAATCTCAGGGTGGTCTGCTGGGTGTTGGTGAATCTGGTTTCGTTGAAGAATCTAACCTGTGGACCTCTTCTCAGCTGTCTCTGACCAACGACGACCTGAACCCGAACGTTCGTGACCCGATCGTTAAACAGCTGGAAGTTTCTTCTGGTATCAAACAGTAAtaatgctagcataaaacgaaaggcccagtctttcgactgagcctttcgttttatttGTAA |
| *ΔlacZ*::*T7 RNAP* | ATGAACACGATTAACATCGCTAAGAACGACTTCTCTGACATCGAACTGGCTGCTATCCCGTTCAACACTCTGGCTGACCATTACGGTGAGCGTTTAGCTCGCGAACAGTTGGCCCTTGAGCATGAGTCTTACGAGATGGGTGAAGCACGCTTCCGCAAGATGTTTGAGCGTCAACTTAAAGCTGGTGAGGTTGCGGATAACGCTGCCGCCAAGCCTCTCATCACTACCCTACTCCCTAAGATGATTGCACGCATCAACGACTGGTTTGAGGAAGTGAAAGCTAAGCGCGGCAAGCGCCCGACAGCCTTCCAGTTCCTGCAAGAAATCAAGCCGGAAGCCGTAGCGTACATCACCATTAAGACCACTCTGGCTTGCCTAACCAGTGCTGACAATACAACCGTTCAGGCTGTAGCAAGCGCAATCGGTCGGGCCATTGAGGACGAGGCTCGCTTCGGTCGTATCCGTGACCTTGAAGCTAAGCACTTCAAGAAAAACGTTGAGGAACAACTCAACAAGCGCGTAGGGCACGTCTACAAGAAAGCATTTATGCAAGTTGTCGAGGCTGACATGCTCTCTAAGGGTCTACTCGGTGGCGAGGCGTGGTCTTCGTGGCATAAGGAAGACTCTATTCATGTAGGAGTACGCTGCATCGAGATGCTCATTGAGTCAACCGGAATGGTTAGCTTACACCGCCAAAATGCTGGCGTAGTAGGTCAAGACTCTGAGACTATCGAACTCGCACCTGAATACGCTGAGGCTATCGCAACCCGTGCAGGTGCGCTGGCTGGCATCTCTCCGATGTTCCAACCTTGCGTAGTTCCTCCTAAGCCGTGGACTGGCATTACTGGTGGTGGCTATTGGGCTAACGGTCGTCGTCCTCTGGCGCTGGTGCGTACTCACAGTAAGAAAGCACTGATGCGCTACGAAGACGTTTACATGCCTGAGGTGTACAAAGCGATTAACATTGCGCAAAACACCGCATGGAAAATCAACAAGAAAGTCCTAGCGGTCGCCAACGTAATCACCAAGTGGAAGCATTGTCCGGTCGAGGACATCCCTGCGATTGAGCGTGAAGAACTCCCGATGAAACCGGAAGACATCGACATGAATCCTGAGGCTCTCACCGCGTGGAAACGTGCTGCCGCTGCTGTGTACCGCAAGGACAAGGCTCGCAAGTCTCGCCGTATCAGCCTTGAGTTCATGCTTGAGCAAGCCAATAAGTTTGCTAACCATAAGGCCATCTGGTTCCCTTACAACATGGACTGGCGCGGTCGTGTTTACGCTGTGTCAATGTTCAACCCGCAAGGTAACGATATGACCAAAGGACTGCTTACGCTGGCGAAAGGTAAACCAATCGGTAAGGAAGGTTACTACTGGCTGAAAATCCACGGTGCAAACTGTGCGGGTGTCGATAAGGTTCCGTTCCCTGAGCGCATCAAGTTCATTGAGGAAAACCACGAGAACATCATGGCTTGCGCTAAGTCTCCACTGGAGAACACTTGGTGGGCTGAGCAAGATTCTCCGTTCTGCTTCCTTGCGTTCTGCTTTGAGTACGCTGGGGTACAGCACCACGGCCTGAGCTATAACTGCTCCCTTCCGCTGGCGTTTGACGGGTCTTGCTCTGGCATCCAGCACTTCTCCGCGATGCTCCGAGATGAGGTAGGTGGTCGCGCGGTTAACTTGCTTCCTAGTGAAACCGTTCAGGACATCTACGGGATTGTTGCTAAGAAAGTCAACGAGATTCTACAAGCAGACGCAATCAATGGGACCGATAACGAAGTAGTTACCGTGACCGATGAGAACACTGGTGAAATCTCTGAGAAAGTCAAGCTGGGCACTAAGGCACTGGCTGGTCAATGGCTGGCTTACGGTGTTACTCGCAGTGTGACTAAGCGTTCAGTCATGACGCTGGCTTACGGGTCCAAAGAGTTCGGCTTCCGTCAACAAGTGCTGGAAGATACCATTCAGCCAGCTATTGATTCCGGCAAGGGTCTGATGTTCACTCAGCCGAATCAGGCTGCTGGATACATGGCTAAGCTGATTTGGGAATCTGTGAGCGTGACGGTGGTAGCTGCGGTTGAAGCAATGAACTGGCTTAAGTCTGCTGCTAAGCTGCTGGCTGCTGAGGTCAAAGATAAGAAGACTGGAGAGATTCTTCGCAAGCGTTGCGCTGTGCATTGGGTAACTCCTGATGGTTTCCCTGTGTGGCAGGAATACAAGAAGCCTATTCAGACGCGCTTGAACCTGATGTTCCTCGGTCAGTTCCGCTTACAGCCTACCATTAACACCAACAAAGATAGCGAGATTGATGCACACAAACAGGAGTCTGGTATCGCTCCTAACTTTGTACACAGCCAAGACGGTAGCCACCTTCGTAAGACTGTAGTGTGGGCACACGAGAAGTACGGAATCGAATCTTTTGCACTGATTCACGACTCCTTCGGTACCATTCCGGCTGACGCTGCGAACCTGTTCAAAGCAGTGCGCGAAACTATGGTTGACACATATGAGTCTTGTGATGTACTGGCTGATTTCTACGACCAGTTCGCTGACCAGTTGCACGAGTCTCAATTGGACAAAATGCCAGCACTTCCGGCTAAAGGTAACTTGAACCTCCGTGACATCTTAGAGTCGGACTTCGCGTTCGCGTAA |

**Table S5. Sequences of modified sites in the *E. coli* chromosome.**

| **Expt**  **No.** | **Host strain** | **Genomic target** | **Guide RNA** | **Homologous arms size (bp)** | **Helper plasmid** | **Donor plasmid** | **aTc (ng/µl)** | **Integration efficiency (%)** | **CFU_arabinose_**  **/CFU_glucose_** |
| --- | --- | --- | --- | --- | --- | --- | --- | --- | --- |
| 1 | MG1655 | *pyrF* | crRNA-*pyrF* | 500 | p46Cpf1 | pTc-P | 0 | 2.8 ± 3.9 | 0.51 ± 0.28 |
| 2 | MG1655 | *pyrF* | crRNA-*pyrF* | 500 | p46Cpf1 | pTc-P | 40 | 77.8 ± 10.4 | 0.15 ± 0.08 |
| 3 | MG1655 | *pyrF* | crRNA-*pyrF* | 500 | p46Cpf1 | pTc-P | 80 | 80.6 ± 17.1 | 0.12 ± 0.02 |
| 4 | MG1655 | *pyrF* | crRNA-*pyrF* | 500 | p46Cpf1-OP1 | pTc-P | 80 | 97.2 ± 4.8 | 0.45 ± 0.39 |
| 5 | MG1655 | *pyrF* | crRNA-*pyrF* | 500 | p46Cpf1-OP2 | pTc-P | 80 | 100.0 ± 0.0 | 0.96 ± 0.51 |
| 6 | MG1655 | *pyrF* | crRNA-*pyrF* | 50 | p46Cpf1 | pTc-P-50 | 80 | 0 | 0.28 ± 0.25 |
| 7 | MG1655 | *pyrF* | crRNA-*pyrF* | 100 | p46Cpf1 | pTc-P-100 | 80 | 16.7 ± 6.8 | 0.74 ± 0.27 |
| 8 | MG1655 | *galK* | crRNA-*galK* | 500 | p46Cpf1 | pTc-G | 80 | 97.2 ± 4.8 | 0.75 ± 0.19 |
| 9 | MG1655 | *galK* | crRNA-*galK2* | 500 | p46Cpf1 | pTc-G2 | 80 | 94.4 ± 4.0 | 0.41 ± 0.16 |
| 10 | MG1655 | *araD* | crRNA-*araD* | 500 | p46Cpf1 | pTc-A | 80 | 19.5 ± 3.9 | 0.73 ± 0.20 |
| 11 | MG1655 | *araD* | crRNA-*araD2* | 500 | p46Cpf1 | pTc-A2 | 80 | 86.1 ± 4.0 | 0.27 ± 0.13 |
| 12 | MG1655 | *araD* | crRNA-*araD2* | 500 | p46Cpf1-OP2 | pTc-A2 | 80 | 94.4 ± 4.0 | 0.85 ± 0.14 |
| 13 | MG1655 | *galK* | crRNA-*galK* | 500 | p46Cpf1-OP2 | pTc-G | 80 | 100.0 ± 0.0 | 0.26 ± 0.06 |
| 14 | MG1655 | *lacZ* | crRNA-*lacZ* | 500 | p46Cpf1-OP2 | pTc-L | 80 | 97.2 ± 4.8 | 0.74 ± 0.17 |
| 15 | MG1655 | *mutS* | crRNA-*mutS* | 500 | p46Cpf1-OP2 | pTc-M | 80 | 97.2 ± 4.8 | 0.27 ± 0.07 |
| 16 | MG1655 | *galK&lacZ* | crRNA-*galK&* crRNA-*lacZ* | 500 | p46Cpf1-OP2 | pTc-GL | 80 | 52.8 ± 12.7 | 0.45 ± 0.29 |
| 17 | MG1655 | *galK&pyrF* | crRNA-*galK&* crRNA-*pyrF* | 500 | p46Cpf1-OP2 | pTc-GP | 80 | 41.7 ± 25.0 | 0.29 ± 0.11 |
| 18 | MG1655 | *lacZ&pyrF* | crRNA-*lacZ&*  crRNA-*pyrF* | 500 | p46Cpf1-OP2 | pTc-PL | 80 | 94.5 ± 3.9 | 0.78 ± 0.24 |
| 19 | MG1655 | *lacZ&pyrF* | crRNA-*lacZ-pyrF* | 500 | p46Cpf1-OP2 | pTc-array-LP | 80 | 97.2 ± 3.9 | 0.63 ± 0.11 |
| 20 | MG1655 | *lacZ&pyrF* | crRNA-*pyrF*-*lacZ* | 500 | p46Cpf1-OP2 | pTc-array-PL | 80 | 91.7 ± 6.8 | 0.61 ± 0.13 |
| 21 | MG1655 | *galK, lacZ&pyrF* | crRNA-*galK,* crRNA-*lacZ&* crRNA-*pyrF* | 500 | p46Cpf1-OP2 | pTc-GLP | 80 | 19.4 ± 14.2 | 0.33 ± 0.15 |
| 23 | MG1655 | *pyrF* | sgRNA-*pyrF* | 500 | p46Cas9 | pTs-P | 80 | 100.0 ± 0.0 | -- |
| 24 | MG1655 | *lacZ&pyrF* | sgRNA-*lacZ&*  sgRNA-*pyrF* | 500 | p46Cas9 | pTs-PL | 80 | 97.2 ± 3.9 | 0.72 ± 0.11 |
| 25 | MG1655 | *galK, lacZ&pyrF* | sgRNA-*galK,* sgRNA-*lacZ&* sgRNA-*pyrF* | 500 | p46Cas9 | pTs-GLP | 80 | 16.7 ± 6.8 | 0.79 ± 0.31 |
| 26 | MG1655 | *torS* | crRNA-*torS* | 500 | p46Cpf1-OP2 | pTc-torS-p103-hem1 | 80 | -- | -- |
| 27 | MG1655 | *lacZ&torS* | crRNA-*lacZ&* crRNA-*torS* | 500 | p46Cpf1-OP2 | pTc-lacZ-T7RNAP-torS-pT7-hem1 | 80 | -- | -- |
| 28 | *H. bluphagenesis* TD01 | *prpC* | crRNA-*prpC* | 500 | pTD-Cas12a | pTtd-prpC | -- | 87.5 | -- |
| 29 | *H. bluphagenesis* TD01 | *prpC* | crRNA-*prpC* | 500 | pTD-Cas12a | pTtd-prpC-PM | -- | 50.0 | -- |

**Table S6. Genome editing results of the two-plasmid system.** CFU_arabinose_, colony number of forming units on plates with arabinose; CFU_glucose_, colony number of forming units on plates with glucose.


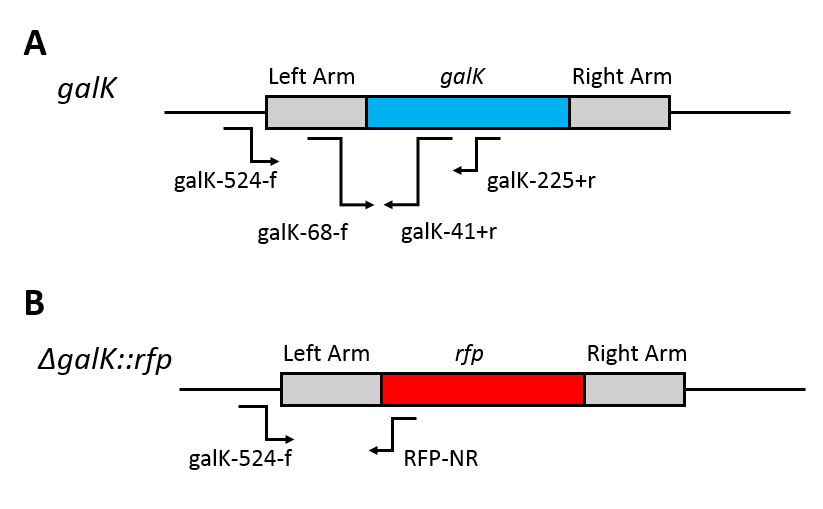


**Figure S1. Schematic diagrams of genotypes and relevant primers for genotyping. (A)** The *galK* locus of wild-type cells. **(B)** The modified *galK* locus with *rfp* gene inserted between the left and right homology arms. When amplifying the genomic locus using the primer pair galK-524-f/galK-225+r (AX042/AX044) or galK-68-f/galK-41+r (AX045/AX046), only wild-type cells produced PCR product (749 bp or 109 bp). When using the primer pair galK-524-f/RFP-NR (AX042/AX043), only those cells which had incorporated the heterologous *rfp* gene produced PCR product (552 bp).


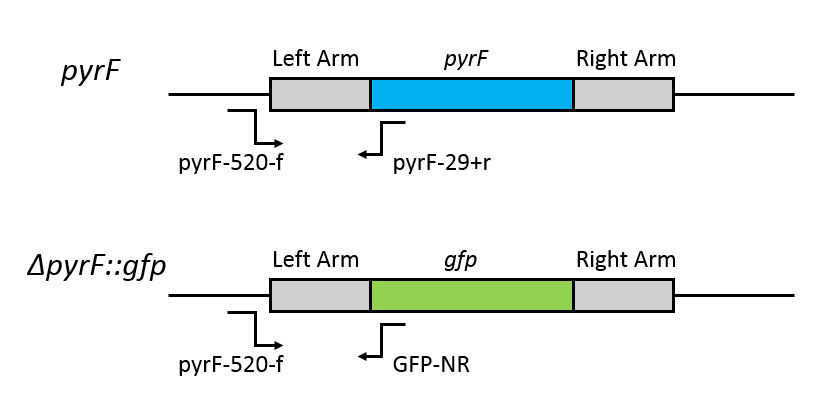


**A**


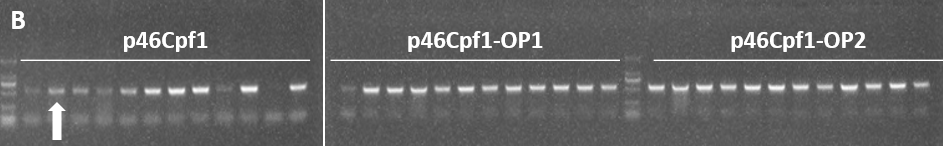


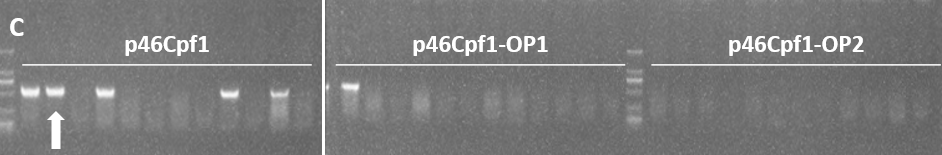


**Figure S2. Results of genotyping PCR when performing gene insertions at the *pyrF* locus with different helper plasmids. (A)** Schematic diagrams of genotypes and relevant primers for genotyping at the *pyrF* locus. **(B)** The donor plasmid pTc-P and different helper plasmids (from left, p46Cpf1, p46Cpf1-OP2 and p46Cpf1-OP2) were used for recombination. The primer pair of pyrF-520-f/GFP-NR (AX052/AX053) was used for genotyping PCR. If a colony contained cells with the modified *pyrF* locus (Δ*pyrF::gfp*), a product of 545 bp would be amplified. **(C)** The tested colonies were the same as those in Fig. S2B. The primer pair of pyrF-520-f/pyrF-29+r (AX052/AX054) was used. If a colony contained cells with the wild-type *pyrF* locus, a product of 549 bp would be amplified. The arrows show the genotyping results of a colony which was composed of both modified and wild-type cells.


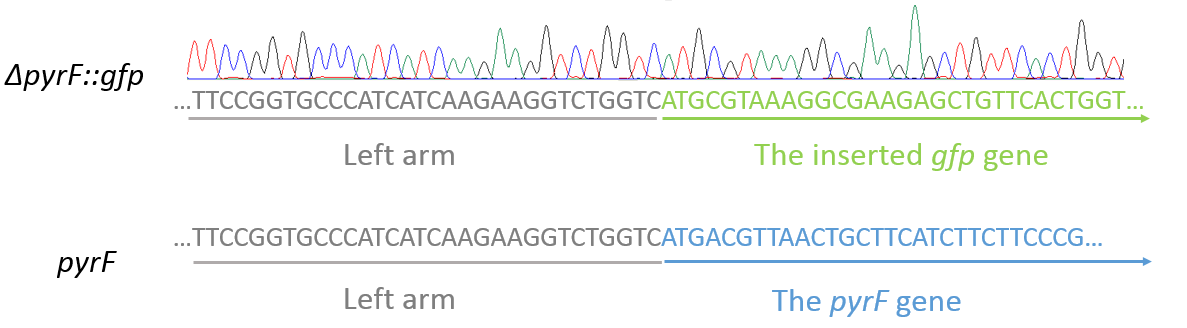


**Figure S3 An example of the DNA sequencing results of the modified genome.** Gene insertion was performed at the *pyrF* locus with p46Cpf1-OP2 and pTc-P, and one of the resulting modified clones was verified by DNA sequencing to confirm the integration of the heterologous gene. The primer pair of AX066/AX052 was used to amplify the modified *pyrF* locus (Δ*pyrF::gfp*).

**
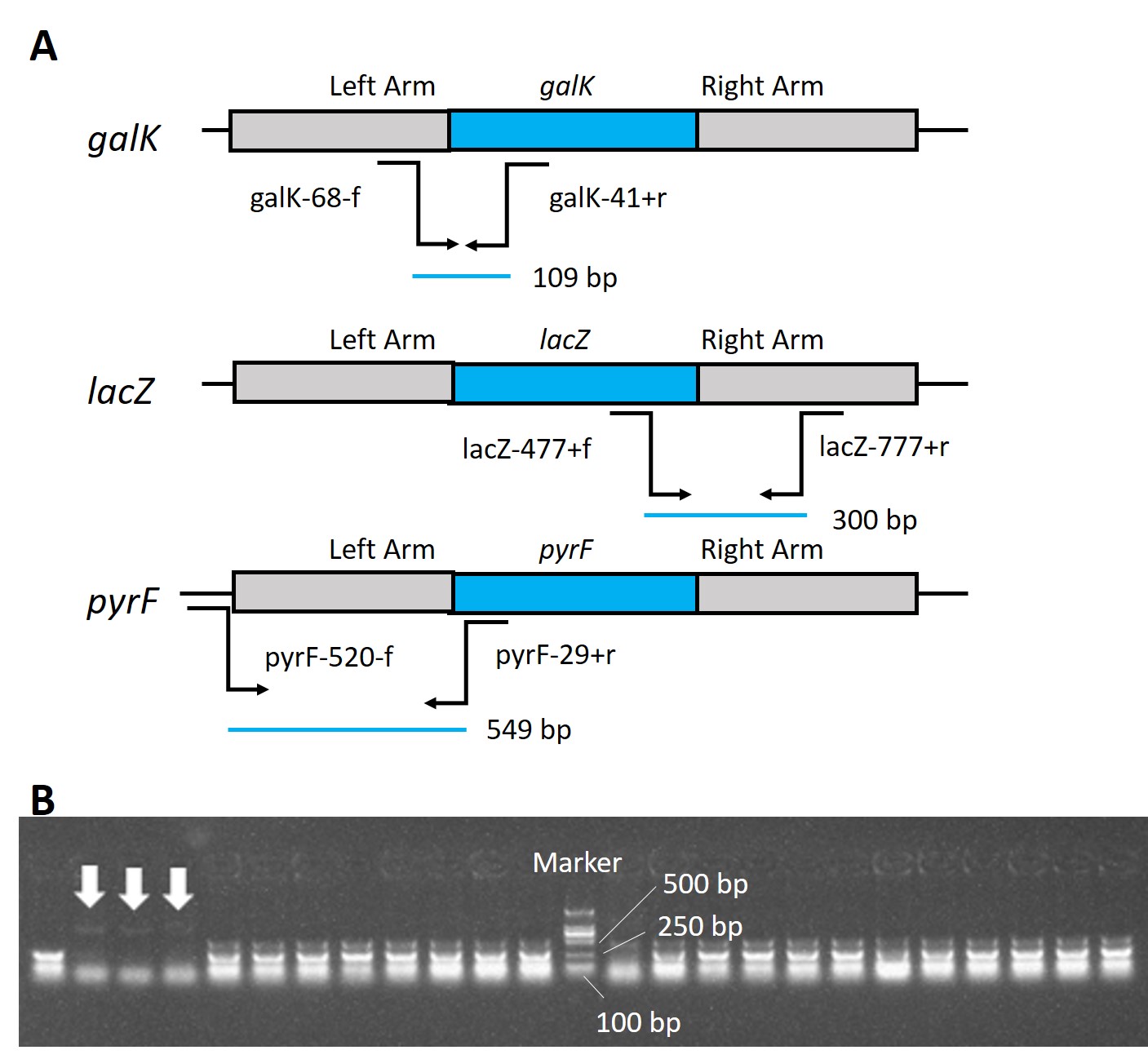
**

**Figure S4. Genotyping PCR when performing gene insertions at three loci simultaneously using the helper plasmid p46Cpf1-OP2. (A)** A sketch map of the relevant primers for genotyping at the 3 loci. **(B)** Results of genotyping PCR. PCR products were separated on a 1.5% agarose gel. Primer pairs of galK-68-f/galK-41+r (AX045/AX046), lacZ-477+f/lacZ-777+r (AX050/AX051) and pyrF-520-f/pyrF-29+r (AX052/AX054) (Table S3) were used simultaneously. The primer pair AX045/AX046 was used to amplify wild-type *galK* locus with a product of 109 bp; the primer pair AX050/AX051 was used to amplify wild-type *galK* locus with a product of 300 bp; the primer pair AX052/AX054 was used to amplify wild-type *pyrF* locus with a product of 549 bp. Thus, PCR products of wild-type cells were a blend of the three binds. Arrows showed colonies that were confirmed to be modified at all the 3 loci because their PCR products were free of binds at 109 bp, 300 bp or 549 bp.

**
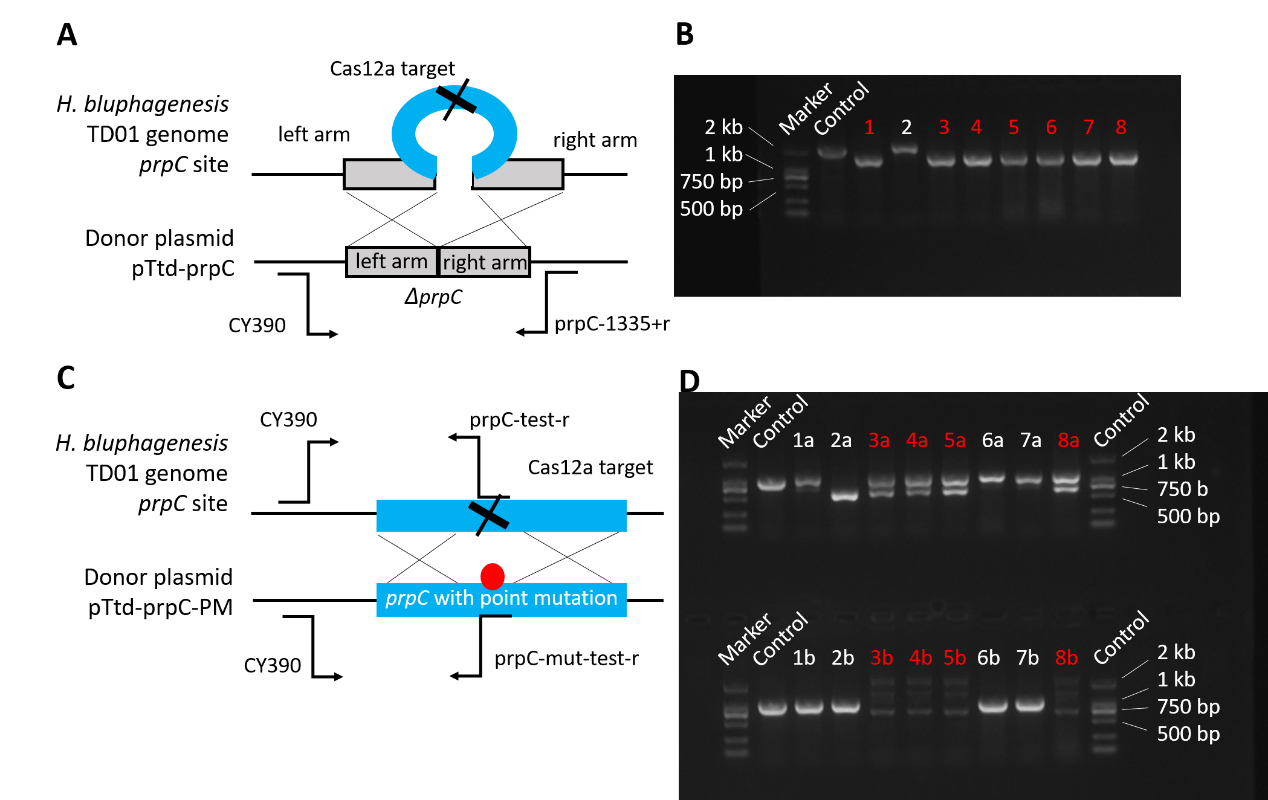
**

**Figure S5. Genotyping PCR for genomic modifications in *H. bluephagenesis.* (A)** A sketch map of recombination between the donor DNA on pTtd-prpC and the *prpC* locus. **(B)** Results of genotyping PCR when using pTtd-prpC. PCR products were separated on a 1.5% agarose gel. The primer pair of CY390/prpC-1335+r (Table S3) was used. The wild-type *prpC* locus was amplified with a product of 2055 bp, while the modified *prpC* locus with a product of 1397 bp. Lanes 1 and 3~8 showed colonies that were confirmed to be modified. **(C)** A sketch map of recombination between the donor DNA on pTtd-prpC-PM and the *prpC* locus. **(D)** Results of genotyping PCR when using pTtd-prpC-PM. PCR products were separated on a 1.5% agarose gel. The primer pairs of CY390/prpC-mut-test-r (upper half) and CY290/prprC-test-r (lower half) (Table S3) were used. The wild-type *prpC* locus was amplified by CY290/prprC-test-r with a product of 1032 bp, while the modified *prpC* locus was amplified by CY290/prprC-mut-test-r with a product of 1032 bp. Lanes 3~5 and 8 showed colonies that were confirmed to be modified.
